# Quinine di­hydro­chloride hemihydrate

**DOI:** 10.1107/S2414314621004065

**Published:** 2021-04-23

**Authors:** Grace I. Anderson, Sophia Bellia, Matthias Zeller, Patrick C. Hillesheim, Arsalan Mirjafari

**Affiliations:** a Department of Chemistry and Physics, Florida Gulf Coast University, 10501 FGCU Blvd. South, Fort Myers, FL, 33965, USA; b Ave Maria University, Department of Chemistry and Physics, 5050 Ave Maria Blvd, Ave Maria FL, 34142, USA; c Purdue University, Department of Chemistry, 560 Oval Drive, West Lafayette, Indiana USA, 47907, USA; Howard University, USA

**Keywords:** crystal structure, hydrogen bonding, ionic compound

## Abstract

Numerous non-covalent inter­actions link together discrete mol­ecules in the crystal structure of the title compound, 2C_20_H_26_N_2_O_2_^2+^·4Cl^−^·H_2_O {systematic name: 4-[(5-ethenyl-1-azonia­bicyclo­[2.2.2]octan-2-yl)(hy­droxy)meth­yl]-6-meth­oxy­quinolin-1-ium dichloride hemihydrate}. A combination of hydrogen bonding between acidic H atoms and the anions in the asymmetric unit forms a portion of the observed hydrogen-bonded network. π–π inter­actions between the aromatic portions of the cation appear to play a role in the formation of the long-range ordering. One ethyl­ene double bond was found to be disordered. The disorder extends to the neighboring carbon and hydrogen atoms.

## Abstract 2

The title compound is a di­hydro­chloride salt with discrete ionic moieties linked together by extensive hydrogen bonding. Serendipitous water in the lattice aids with the formation of these hydrogen-bonding networks.

## Graphical abstract

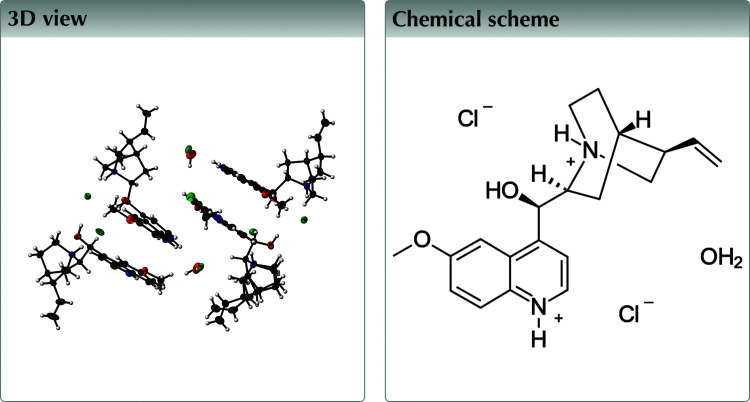

## Structure description

**Figure 1:**
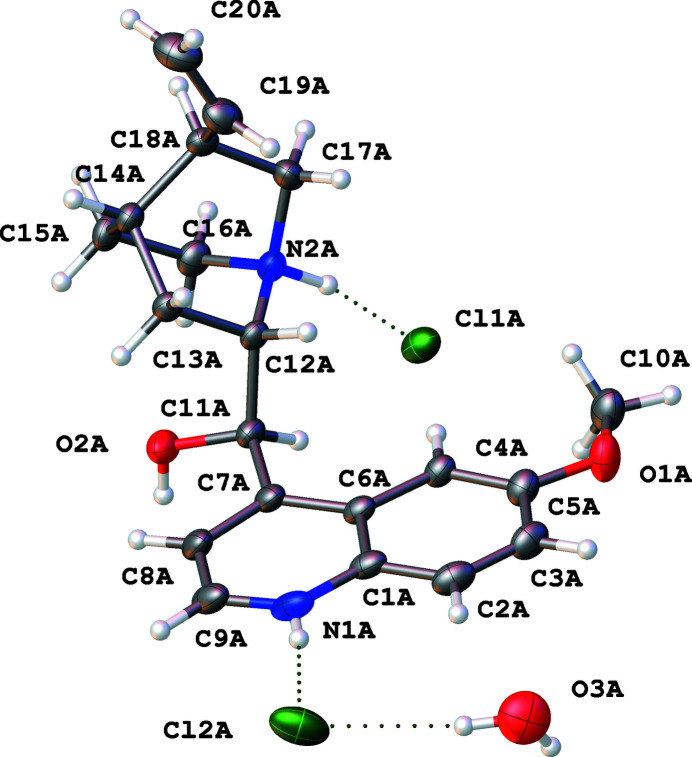
Labeling scheme for the cationic portion of the structure. Four distinct dicationic moieties are in the asymmetric unit, labeled using suffixes *A*, *B*, *C* and *D*.

**Figure 2:**
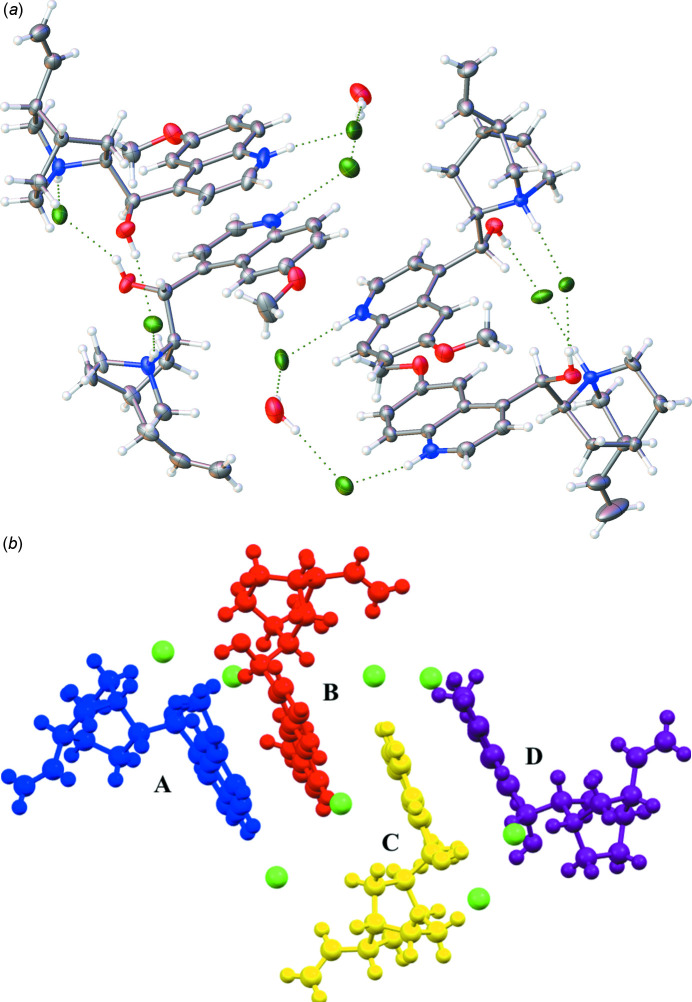
(*a*) The asymmetric unit of the title compound shown with 50% probability ellipsoids. Disorder is omitted for clarity. (*b*) The asymmetric unit of the title compound colored to define the distinct cation pairs *A* and *B* (blue and red) and *C* and *D* (yellow and purple) discussed in the manuscript.

The title salt (Fig. 1 ▸) crystallizes in the *P*1 space group with four distinct cation–anion pairs in the asymmetric unit and two water mol­ecules. The four compounds form discrete pairs wherein two of the dications are linked together by extensive hydrogen bonding involving the chloride anions. Specifically, moieties *A* and *B* are linked through hydrogen-bonding networks while *C* and *D* are joined to form a second hydrogen-bonded moiety. Within these moiety pairs, distinct hydrogen-bonded chains facilitate the observed packing (Table 1 ▸, Fig. 2 ▸). For example, hydrogen bonding is observed between the protonated quinuclidine nitro­gen and the alcohol group, effectively bridged by a chloride anion making a N—H⋯Cl⋯H—O linkage. This inter­action is reciprocal between the moieties.

The second major hydrogen-bonded chain is between the protonated quinolinium N—H moieties of the linked pairs. This hydrogen bonding involves a serendipitous water mol­ecule helping to bridge the discrete N—H⋯Cl inter­actions, effectively forming an N—H⋯Cl⋯H—O—H⋯Cl⋯H—N chain.

**Figure 3:**
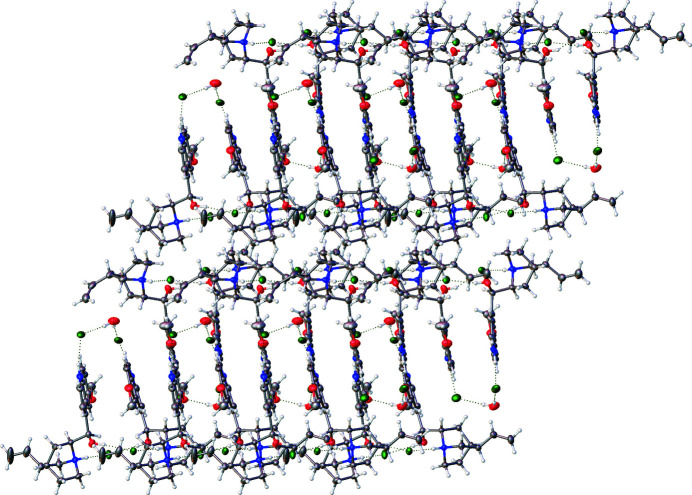
Packing diagram of the title compound viewed from the (110) plane.

**Figure 4:**
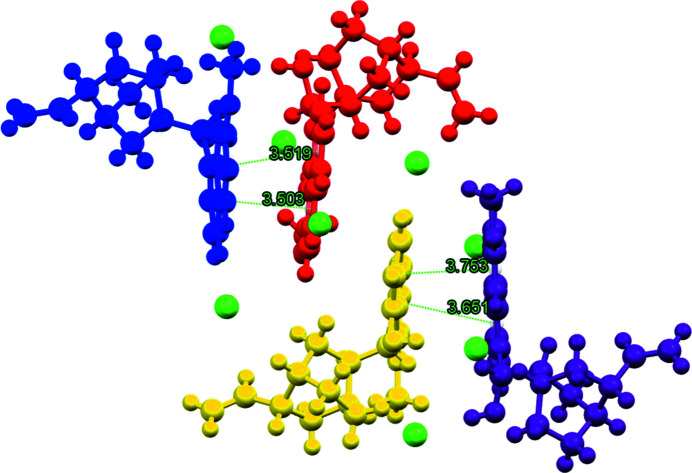
π–π inter­actions between cationic moieties in the asymmetric unit. Centroid-centroid distances are shown in green.

The distinct dication pairs, that is *A* & *B* and *C* & *D*, are also linked through π–π stacking inter­actions. Centroid–centroid distances ranging from approximately 3.50 to 3.75 Å are observed between the discrete aromatic rings of the quinolinium ring systems. These π–π inter­actions are perhaps best seen when examining the packing diagram in Fig. 3 ▸ and the centroid–centroid distances shown in Fig. 4 ▸.

## Synthesis and crystallization

The chloride salt of the quinine was formed *via* neutralization of the quinine with hydro­chloric acid. Hydro­chloric acid (2 equiv., 37% *w*/*w*) was added to a solution of quinine (1 equiv.) in 25 ml of aceto­nitrile while stirring. A white crystalline solid formed after the flask is left standing for 4 h. After isolating the product *via* vacuum filtration, it was recrystallized *via* the vapor diffusion method. 1 ml of a saturated solution of the salt was placed in a 5 ml glass vial, methyl *tert*-butyl ether was added to the large vial as the anti­solvent and the larger vial was tightly sealed. White crystals formed after a week at room temperature.

## Refinement

Crystal data, data collection and structure refinement details are summarized in Table 2 ▸. One ethyl­ene double bond was found to be disordered. The disorder extends to the neighboring carbon and hydrogen atoms. The two disordered moieties were restrained to have a similar geometry as another not disordered equivalent group. *U^ij^* components of ADPs for disordered atoms closer to each other than 2.0 Å were restrained to be similar (e.s.d. 0.01 Å^2^). Subject to these conditions the occupancy ratio refined to 0.505 (9):0.495 (9).

## Supplementary Material

Crystal structure: contains datablock(s) I, global. DOI: 10.1107/S2414314621004065/bv4036sup1.cif

Structure factors: contains datablock(s) I. DOI: 10.1107/S2414314621004065/bv4036Isup2.hkl

CCDC reference: 2077945

Additional supporting information:  crystallographic information; 3D view; checkCIF report

## Figures and Tables

**Table 1:** Hydrogen-bond geometry (Å, °)

*D*—H⋯*A*	*D*—H	H⋯*A*	*D*⋯*A*	*D*—H⋯*A*
O2*A*—H2*OA*⋯Cl1*B*	0.84	2.23	3.0540 (18)	166
O3*A*—H3*E*⋯Cl2*A*	0.89 (2)	2.42 (3)	3.303 (3)	172 (4)
O3*A*—H3*F*⋯Cl2*B*	0.86 (2)	2.42 (3)	3.275 (3)	171 (4)
N1*A*—H1*NA*⋯Cl2*A*	0.88	2.06	2.935 (2)	173
N2*A*—H2*NA*⋯Cl1*A*	1.00	2.04	3.0226 (18)	166
C2*A*—H2*A*⋯O3*A*	0.95	2.51	3.414 (3)	160
C5*A*—H5*A*⋯Cl1*A*	0.95	2.93	3.872 (2)	172
C9*A*—H9*A*⋯Cl2*C* ^i^	0.95	2.98	3.879 (3)	158
C11*A*—H11*A*⋯Cl1*A*	1.00	2.76	3.608 (2)	143
C13*A*—H13*B*⋯Cl2*D* ^ii^	0.99	2.95	3.787 (2)	142
C15*A*—H15*A*⋯O2*C* ^iii^	0.99	2.63	3.526 (3)	151
C16*A*—H16*A*⋯O2*A*	0.99	2.38	2.948 (3)	116
O2*B*—H2*OB*⋯Cl1*A*	0.84	2.31	3.0834 (17)	154
N1*B*—H1*NB*⋯Cl2*B*	0.88	2.15	3.020 (2)	168
N2*B*—H2*NB*⋯Cl1*B*	1.00	2.03	3.0110 (18)	168
C2*B*—H2*B*⋯Cl2*B*	0.95	2.72	3.479 (2)	137
C9*B*—H9*B*⋯Cl2*D* ^iv^	0.95	2.73	3.534 (2)	142
C10*B*—H10*D*⋯Cl1*B*	0.98	2.86	3.606 (3)	133
C12*B*—H12*B*⋯Cl2*C*	1.00	2.90	3.646 (2)	132
C13*B*—H13*D*⋯Cl2*C*	0.99	2.78	3.559 (2)	136
C15*B*—H15*D*⋯O2*B*	0.99	2.51	3.041 (10)	113
C16*B*—H16*C*⋯O2*B*	0.99	2.45	2.942 (15)	110
C16*B*—H16*D*⋯Cl1*C* ^v^	0.99	2.88	3.606 (16)	131
C17*B*—H17*D*⋯O3*C*	0.99	2.51	3.492 (3)	173
C16*E*—H16*E*⋯Cl1*C* ^v^	0.99	2.77	3.483 (17)	129
C16*E*—H16*F*⋯O2*B*	0.99	2.52	3.066 (17)	115
C17*E*—H17*F*⋯O3*C*	0.99	2.51	3.492 (3)	173
O2*C*—H2*OC*⋯Cl1*D*	0.84	2.18	3.0042 (16)	166
O3*C*—H3*G*⋯Cl2*C*	0.88 (3)	2.51 (3)	3.381 (3)	171 (4)
O3*C*—H3*H*⋯Cl2*D*	0.89 (3)	2.41 (3)	3.294 (3)	168 (4)
N1*C*—H1*NC*⋯Cl2*C*	0.88	2.11	2.986 (2)	172
N2*C*—H2*NC*⋯Cl1*C*	1.00	2.02	3.0095 (18)	169
C2*C*—H2*C*⋯O3*C*	0.95	2.45	3.351 (3)	159
C5*C*—H5*C*⋯Cl1*C*	0.95	2.90	3.827 (2)	165
C11*C*—H11*C*⋯Cl1*C*	1.00	2.79	3.630 (2)	142
C15*C*—H15*G*⋯Cl1*A* ^vi^	0.99	2.97	3.750 (2)	136
C15*C*—H15*H*⋯O2*C*	0.99	2.50	3.038 (3)	114
C16*C*—H16*H*⋯Cl1*A* ^vi^	0.99	2.93	3.717 (2)	137
C17*C*—H17*G*⋯Cl1*D* ^i^	0.99	2.69	3.411 (2)	130
O2*D*—H2*OD*⋯Cl1*C*	0.84	2.21	3.0442 (17)	172
N1*D*—H1*ND*⋯Cl2*D*	0.88	2.19	3.054 (2)	166
N2*D*—H2*ND*⋯Cl1*D*	1.00	1.99	2.9885 (18)	177
C2*D*—H2*D*⋯Cl2*D*	0.95	2.95	3.664 (2)	134
C5*D*—H5*D*⋯Cl1*D*	0.95	2.93	3.864 (2)	167
C9*D*—H9*D*⋯Cl2*B* ^vii^	0.95	2.79	3.488 (2)	131
C10*D*—H10*K*⋯Cl2*A* ^viii^	0.98	2.92	3.627 (3)	130
C11*D*—H11*D*⋯Cl1*D*	1.00	2.89	3.725 (2)	141
C13*D*—H13*G*⋯O3*A* ^ix^	0.99	2.43	3.340 (3)	153
C16*D*—H16*I*⋯O2*D*	0.99	2.41	3.080 (3)	124
C16*D*—H16*J*⋯Cl1*B* ^x^	0.99	2.82	3.653 (2)	143
C17*D*—H17*J*⋯Cl1*C* ^viii^	0.99	2.74	3.513 (2)	135

Symmetry codes: (i) 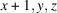 ; (ii) 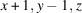 ; (iii) 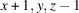 ; (iv) 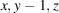 ; (v) 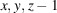 ; (vi) 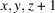 ; (vii) 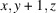 ; (viii) 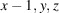 ; (ix) 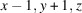 ; (x) 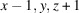 .

**Table 2:** Experimental details

Crystal data	
Chemical formula	2C_20_H_26_N_2_O_2_^2+^·4Cl^−^·H_2_O
*M*_r_	812.67
Crystal system, space group	Triclinic, *P*1
Temperature (K)	150
*a*, *b*, *c* (Å)	9.5944 (3), 12.7606 (4), 18.0312 (6)
α, β, γ (°)	73.9951 (18), 79.1246 (18), 75.2203 (17)
*V* (Å^3^)	2035.09 (12)
*Z*	2
Radiation type	Mo *K*α
μ (mm^−1^)	0.34
Crystal size (mm)	0.44 × 0.29 × 0.20

Data collection	
Diffractometer	Bruker AXS D8 Quest with PhotonII charge-integrating pixel array detector (CPAD)
Absorption correction	Multi-scan (*SADABS*; Krause *et al.*, 2015 ▸)
*T*_min_, *T*_max_	0.678, 0.747
No. of measured, independent and observed [*I* > 2σ(*I*)] reflections	120029, 30500, 24850
*R*_int_	0.050
(sin θ/λ)_max_ (Å^−1^)	0.771

Refinement	
*R*[*F* ^2^ > 2σ(*F* ^2^)], *wR*(*F* ^2^), *S*	0.038, 0.090, 1.02
No. of reflections	30500
No. of parameters	1030
No. of restraints	233
H-atom treatment	H atoms treated by a mixture of independent and constrained refinement
Δρ_max_, Δρ_min_ (e Å^−3^)	0.49, −0.41
Absolute structure	Flack *x* determined using 10201 quotients [(*I* ^+^)−(*I* ^−^)]/[(*I* ^+^)+(*I* ^−^)] (Parsons *et al.*, 2013 ▸)
Absolute structure parameter	−0.015 (9)

Computer programs: *APEX3* and *SAINT* (Bruker, 2020 ▸), *SHELXT* (Sheldrick, 2015*a* ▸), *SHELXL2018/3* (Sheldrick, 2015*b* ▸), *shelXle* (Hübschle *et al.*, 2011 ▸), *OLEX2* (Dolomanov *et al.*, 2009 ▸), *publCIF* (Westrip, 2010 ▸), CSD (Groom *et al.*, 2016 ▸) and *enCIFer* (Allen *et al.*, 2004 ▸).

## full crystallographic data

### Crystal data

**Table:**

2C_20_H_26_N_2_O_2_^2+^·4Cl^−^·H_2_O	*Z* = 2
*M**_r_* = 812.67	*F*(000) = 860
Triclinic, *P*1	*D*_x_ = 1.326 Mg m^−^^3^
*a* = 9.5944 (3) Å	Mo *K*α radiation, λ = 0.71073 Å
*b* = 12.7606 (4) Å	Cell parameters from 9741 reflections
*c* = 18.0312 (6) Å	θ = 2.6–32.4°
α = 73.9951 (18)°	µ = 0.34 mm^−^^1^
β = 79.1246 (18)°	*T* = 150 K
γ = 75.2203 (17)°	Plate, colourless
*V* = 2035.09 (12) Å^3^	0.44 × 0.29 × 0.20 mm

### Data collection

**Table:**

Bruker AXS D8 Quest diffractometer with PhotonII charge-integrating pixel array detector (CPAD)	30500 independent reflections
Radiation source: fine focus sealed tube X-ray source	24850 reflections with *I* > 2σ(*I*)
Triumph curved graphite crystal monochromator	*R*_int_ = 0.050
Detector resolution: 7.4074 pixels mm^-1^	θ_max_ = 33.2°, θ_min_ = 2.2°
ω and phi scans	*h* = −14→14
Absorption correction: multi-scan (SADABS; Krause *et al.*, 2015)	*k* = −19→19
*T*_min_ = 0.678, *T*_max_ = 0.747	*l* = −27→27
120029 measured reflections	

### Refinement

**Table:**

Refinement on *F*^2^	Secondary atom site location: difference Fourier map
Least-squares matrix: full	Hydrogen site location: mixed
*R*[*F*^2^ > 2σ(*F*^2^)] = 0.038	H atoms treated by a mixture of independent and constrained refinement
*wR*(*F*^2^) = 0.090	*w* = 1/[σ^2^(*F*_o_^2^) + (0.0421*P*)^2^ + 0.1915*P*] where *P* = (*F*_o_^2^ + 2*F*_c_^2^)/3
*S* = 1.02	(Δ/σ)_max_ = 0.001
30500 reflections	Δρ_max_ = 0.49 e Å^−^^3^
1030 parameters	Δρ_min_ = −0.41 e Å^−^^3^
233 restraints	Absolute structure: Flack *x* determined using 10201 quotients [(*I*^+^)-(*I*^-^)]/[(*I*^+^)+(*I*^-^)] (Parsons *et al.*, 2013)
Primary atom site location: dual	Absolute structure parameter: −0.015 (9)

### Special details

**Table:**

Geometry. All esds (except the esd in the dihedral angle between two l.s. planes) are estimated using the full covariance matrix. The cell esds are taken into account individually in the estimation of esds in distances, angles and torsion angles; correlations between esds in cell parameters are only used when they are defined by crystal symmetry. An approximate (isotropic) treatment of cell esds is used for estimating esds involving l.s. planes.
Refinement. One ethylene double bond was found to be disordered. The disorder extends to the neighboring carbon and hydrogen atoms. The two disordered moieties were restrained to have a similar geometry as another not disordered equivalent group. Uij components of ADPs for disordered atoms closer to each other than 2.0 Angstrom were restrained to be similar. Subject to these conditions the occupancy ratio refined to 0.505 (9) to 0.495 (9).

### Fractional atomic coordinates and isotropic or equivalent isotropic displacement parameters (Å^2^)

**Table:**

	*x*	*y*	*z*	*U*_iso_*/*U*_eq_	Occ. (<1)
Cl1A	0.73620 (6)	0.20342 (5)	0.09724 (3)	0.03224 (11)	
Cl2A	0.98383 (9)	0.24836 (6)	0.59486 (4)	0.04950 (18)	
O1A	0.6617 (2)	−0.00094 (16)	0.35855 (11)	0.0380 (4)	
O2A	0.99946 (19)	0.40649 (13)	0.14679 (11)	0.0336 (4)	
H2OA	0.928698	0.460326	0.150030	0.050*	
O3A	0.7857 (3)	0.0577 (2)	0.66750 (13)	0.0546 (6)	
H3E	0.835 (5)	0.112 (3)	0.652 (3)	0.082*	
H3F	0.695 (3)	0.085 (4)	0.661 (3)	0.082*	
N1A	0.9605 (2)	0.26196 (19)	0.43234 (12)	0.0328 (4)	
H1NA	0.967585	0.251275	0.481960	0.039*	
N2A	1.05718 (19)	0.18930 (15)	0.09162 (10)	0.0240 (3)	
H2NA	0.955420	0.180398	0.095037	0.029*	
C1A	0.8921 (2)	0.1951 (2)	0.41131 (12)	0.0261 (4)	
C2A	0.8315 (2)	0.1140 (2)	0.46910 (13)	0.0315 (5)	
H2A	0.841722	0.103884	0.522160	0.038*	
C3A	0.7586 (3)	0.0503 (2)	0.44887 (13)	0.0312 (5)	
H3A	0.717998	−0.004657	0.487857	0.037*	
C4A	0.7424 (2)	0.06527 (19)	0.36958 (13)	0.0273 (4)	
C5A	0.8043 (2)	0.14162 (18)	0.31157 (12)	0.0237 (4)	
H5A	0.795432	0.149291	0.258647	0.028*	
C6A	0.8819 (2)	0.20905 (18)	0.33190 (12)	0.0224 (4)	
C7A	0.9489 (2)	0.29154 (19)	0.27689 (13)	0.0250 (4)	
C8A	1.0119 (3)	0.3584 (2)	0.30309 (15)	0.0335 (5)	
H8A	1.052243	0.416075	0.266876	0.040*	
C9A	1.0166 (2)	0.3417 (2)	0.38201 (16)	0.0352 (5)	
H9A	1.060216	0.387795	0.399733	0.042*	
C10A	0.6276 (3)	0.0136 (2)	0.28178 (15)	0.0370 (5)	
H10A	0.576570	0.090898	0.262742	0.056*	
H10B	0.565434	−0.037196	0.283174	0.056*	
H10C	0.717669	−0.002845	0.246932	0.056*	
C11A	0.9588 (2)	0.30587 (17)	0.18971 (12)	0.0246 (4)	
H11A	0.863314	0.303472	0.176318	0.029*	
C12A	1.0766 (2)	0.20798 (17)	0.16823 (11)	0.0215 (4)	
H12A	1.064880	0.139046	0.209610	0.026*	
C13A	1.2332 (2)	0.22229 (18)	0.16295 (12)	0.0238 (4)	
H13A	1.231365	0.296580	0.170564	0.029*	
H13B	1.283323	0.164662	0.204301	0.029*	
C14A	1.3155 (2)	0.21105 (17)	0.08285 (12)	0.0237 (4)	
H14A	1.417098	0.220586	0.078983	0.028*	
C15A	1.2376 (2)	0.29936 (19)	0.01932 (13)	0.0287 (4)	
H15A	1.292317	0.293563	−0.032256	0.034*	
H15B	1.233229	0.374622	0.026006	0.034*	
C16A	1.0834 (2)	0.2835 (2)	0.02313 (12)	0.0286 (4)	
H16A	1.011828	0.352865	0.028836	0.034*	
H16B	1.072460	0.265947	−0.025368	0.034*	
C17A	1.1593 (3)	0.08365 (19)	0.07935 (14)	0.0303 (5)	
H17A	1.142274	0.067187	0.031679	0.036*	
H17B	1.141280	0.020918	0.123923	0.036*	
C18A	1.3184 (2)	0.09502 (18)	0.07139 (13)	0.0258 (4)	
H18A	1.364502	0.092161	0.017199	0.031*	
C19A	1.4062 (3)	0.0021 (2)	0.12703 (15)	0.0352 (5)	
H19A	1.357501	−0.029675	0.175840	0.042*	
C20A	1.5464 (3)	−0.0373 (2)	0.1118 (2)	0.0453 (6)	
H20A	1.597968	−0.007064	0.063401	0.054*	
H20B	1.596177	−0.095981	0.149076	0.054*	
Cl1B	0.77406 (5)	0.62591 (5)	0.13660 (3)	0.02989 (11)	
Cl2B	0.44651 (7)	0.13687 (5)	0.63330 (4)	0.03996 (14)	
O1B	0.7630 (2)	0.57884 (17)	0.40286 (11)	0.0385 (4)	
O2B	0.49213 (18)	0.36221 (15)	0.17582 (11)	0.0335 (4)	
H2OB	0.566994	0.312764	0.169304	0.050*	
N1B	0.4529 (2)	0.25198 (15)	0.46257 (12)	0.0283 (4)	
H1NB	0.439682	0.214689	0.511364	0.034*	
N2B	0.4620 (2)	0.62089 (16)	0.13667 (10)	0.0257 (4)	
H2NB	0.560289	0.627392	0.143396	0.031*	
C1B	0.5310 (2)	0.33354 (17)	0.44418 (13)	0.0234 (4)	
C2B	0.5920 (2)	0.35163 (19)	0.50386 (13)	0.0265 (4)	
H2B	0.580120	0.307123	0.555450	0.032*	
C3B	0.6679 (2)	0.4336 (2)	0.48632 (14)	0.0284 (4)	
H3B	0.709789	0.446094	0.525977	0.034*	
C4B	0.6853 (2)	0.50075 (19)	0.40971 (13)	0.0268 (4)	
C5B	0.6314 (2)	0.48204 (18)	0.35001 (13)	0.0246 (4)	
H5B	0.647412	0.525682	0.298445	0.030*	
C6B	0.5514 (2)	0.39653 (17)	0.36641 (12)	0.0221 (4)	
C7B	0.4937 (2)	0.36783 (17)	0.30900 (13)	0.0233 (4)	
C8B	0.4157 (2)	0.28401 (18)	0.33216 (14)	0.0281 (4)	
H8B	0.375076	0.265423	0.294608	0.034*	
C9B	0.3965 (2)	0.22696 (19)	0.40967 (15)	0.0316 (5)	
H9B	0.342646	0.169792	0.424834	0.038*	
C10B	0.7796 (4)	0.6557 (3)	0.32911 (18)	0.0585 (9)	
H10D	0.828986	0.614681	0.289530	0.088*	
H10E	0.837515	0.707307	0.331970	0.088*	
H10F	0.683677	0.698066	0.315186	0.088*	
C11B	0.5169 (2)	0.42593 (18)	0.22295 (13)	0.0249 (4)	
H11B	0.618701	0.437601	0.208714	0.030*	
C12B	0.4094 (2)	0.54002 (17)	0.20860 (11)	0.0220 (4)	
H12B	0.402181	0.572305	0.254162	0.026*	
C13B	0.2552 (2)	0.53338 (18)	0.20066 (13)	0.0243 (4)	
H13C	0.250555	0.454517	0.207865	0.029*	
H13D	0.184571	0.563706	0.241256	0.029*	
C14B	0.2168 (14)	0.5998 (10)	0.1210 (7)	0.031 (2)	0.505 (9)
H14B	0.119691	0.591180	0.114039	0.038*	0.505 (9)
C15B	0.3335 (12)	0.5559 (9)	0.0595 (6)	0.0349 (18)	0.505 (9)
H15C	0.304929	0.592212	0.006925	0.042*	0.505 (9)
H15D	0.344184	0.474355	0.068213	0.042*	0.505 (9)
C16B	0.4783 (14)	0.5818 (12)	0.0656 (9)	0.030 (2)	0.505 (9)
H16C	0.556748	0.513785	0.067588	0.037*	0.505 (9)
H16D	0.504421	0.640002	0.019599	0.037*	0.505 (9)
C17B	0.3633 (3)	0.7332 (2)	0.13301 (14)	0.0328 (5)	0.505 (9)
H17C	0.404081	0.790857	0.091891	0.039*	0.505 (9)
H17D	0.349892	0.753672	0.183411	0.039*	0.505 (9)
C18B	0.2128 (11)	0.7231 (11)	0.1137 (8)	0.037 (2)	0.505 (9)
H18B	0.207229	0.761728	0.057612	0.045*	0.505 (9)
C19B	0.0738 (6)	0.7754 (5)	0.1593 (4)	0.0386 (14)	0.505 (9)
H19B	−0.015260	0.768750	0.147300	0.046*	0.505 (9)
C20B	0.0648 (7)	0.8285 (5)	0.2132 (4)	0.0415 (16)	0.505 (9)
H20C	0.150695	0.837476	0.227417	0.050*	0.505 (9)
H20D	−0.027615	0.857960	0.238082	0.050*	0.505 (9)
C14E	0.2037 (15)	0.6171 (10)	0.1251 (7)	0.032 (2)	0.495 (9)
H14E	0.102063	0.614168	0.121653	0.038*	0.495 (9)
C15E	0.3046 (11)	0.5832 (10)	0.0552 (8)	0.0359 (19)	0.495 (9)
H15E	0.302742	0.505890	0.055307	0.043*	0.495 (9)
H15F	0.270923	0.633330	0.006342	0.043*	0.495 (9)
C16E	0.4573 (14)	0.5904 (13)	0.0594 (10)	0.032 (2)	0.495 (9)
H16E	0.488694	0.648094	0.014477	0.039*	0.495 (9)
H16F	0.524637	0.517929	0.057199	0.039*	0.495 (9)
C17E	0.3633 (3)	0.7332 (2)	0.13301 (14)	0.0328 (5)	0.495 (9)
H17E	0.400873	0.787661	0.087866	0.039*	0.495 (9)
H17F	0.364811	0.757151	0.180568	0.039*	0.495 (9)
C18E	0.2084 (12)	0.7354 (11)	0.1259 (7)	0.0348 (19)	0.495 (9)
H18E	0.181972	0.787197	0.075374	0.042*	0.495 (9)
C19E	0.1064 (7)	0.7771 (5)	0.1925 (4)	0.0322 (12)	0.495 (9)
H19E	0.131328	0.745025	0.243699	0.039*	0.495 (9)
C20E	−0.0121 (7)	0.8533 (5)	0.1840 (4)	0.0444 (17)	0.495 (9)
H20E	−0.040323	0.887158	0.133615	0.053*	0.495 (9)
H20F	−0.070621	0.875258	0.228177	0.053*	0.495 (9)
Cl1C	0.49022 (6)	0.67007 (4)	0.85699 (3)	0.02883 (11)	
Cl2C	0.18968 (7)	0.58081 (6)	0.39094 (3)	0.03816 (13)	
O1C	0.5811 (2)	0.83667 (15)	0.59141 (10)	0.0373 (4)	
O2C	0.35728 (16)	0.38009 (13)	0.81883 (9)	0.0270 (3)	
H2OC	0.270717	0.407543	0.833945	0.041*	
O3C	0.3500 (3)	0.8001 (2)	0.30978 (13)	0.0577 (6)	
H3G	0.299 (5)	0.748 (3)	0.329 (3)	0.087*	
H3H	0.309 (5)	0.859 (3)	0.330 (3)	0.087*	
N1C	0.3053 (2)	0.53830 (17)	0.54154 (12)	0.0309 (4)	
H1NC	0.279318	0.552070	0.494942	0.037*	
N2C	0.63540 (18)	0.43684 (14)	0.84200 (10)	0.0211 (3)	
H2NC	0.599598	0.515834	0.846641	0.025*	
C1C	0.3773 (2)	0.60904 (19)	0.55565 (12)	0.0268 (4)	
C2C	0.4138 (3)	0.6985 (2)	0.49535 (13)	0.0341 (5)	
H2C	0.390143	0.708512	0.444673	0.041*	
C3C	0.4827 (3)	0.7707 (2)	0.50921 (14)	0.0346 (5)	
H3C	0.508930	0.829846	0.467999	0.042*	
C4C	0.5156 (2)	0.75774 (19)	0.58526 (13)	0.0283 (4)	
C5C	0.4820 (2)	0.67017 (17)	0.64559 (12)	0.0230 (4)	
H5C	0.503392	0.662947	0.696336	0.028*	
C6C	0.4152 (2)	0.59089 (17)	0.63155 (12)	0.0226 (4)	
C7C	0.3835 (2)	0.49450 (16)	0.68794 (12)	0.0218 (4)	
C8C	0.3136 (2)	0.42530 (19)	0.66858 (13)	0.0275 (4)	
H8C	0.293259	0.360446	0.705961	0.033*	
C9C	0.2730 (3)	0.4504 (2)	0.59436 (15)	0.0323 (5)	
H9C	0.221851	0.404213	0.581817	0.039*	
C10C	0.6150 (3)	0.8324 (2)	0.66662 (16)	0.0375 (5)	
H10G	0.674144	0.758669	0.687412	0.056*	
H10H	0.524753	0.845618	0.701920	0.056*	
H10I	0.669297	0.889887	0.661835	0.056*	
C11C	0.4237 (2)	0.46536 (16)	0.77001 (12)	0.0211 (3)	
H11C	0.389745	0.533336	0.791390	0.025*	
C12C	0.5895 (2)	0.42714 (16)	0.76887 (11)	0.0198 (3)	
H12C	0.634881	0.478906	0.723969	0.024*	
C13C	0.6551 (2)	0.30703 (17)	0.75933 (12)	0.0228 (4)	
H13E	0.577700	0.273253	0.752452	0.027*	
H13F	0.728511	0.308539	0.712622	0.027*	
C14C	0.7264 (2)	0.23702 (17)	0.83192 (12)	0.0251 (4)	
H14C	0.762040	0.157766	0.827862	0.030*	
C15C	0.6137 (3)	0.24233 (18)	0.90399 (13)	0.0278 (4)	
H15G	0.653049	0.188837	0.950343	0.033*	
H15H	0.524659	0.222384	0.896950	0.033*	
C16C	0.5778 (2)	0.36157 (18)	0.91523 (12)	0.0254 (4)	
H16G	0.471312	0.387352	0.926351	0.030*	
H16H	0.623014	0.363403	0.959750	0.030*	
C17C	0.7987 (2)	0.40983 (19)	0.83467 (14)	0.0277 (4)	
H17G	0.831098	0.432294	0.875869	0.033*	
H17H	0.838956	0.451276	0.783521	0.033*	
C18C	0.8539 (2)	0.28263 (19)	0.84262 (13)	0.0277 (4)	
H18C	0.877849	0.247869	0.896883	0.033*	
C19C	0.9889 (3)	0.2566 (2)	0.78792 (14)	0.0347 (5)	
H19C	0.984881	0.290180	0.734124	0.042*	
C20C	1.1122 (3)	0.1912 (2)	0.80812 (18)	0.0402 (6)	
H20G	1.120824	0.156001	0.861323	0.048*	
H20H	1.192929	0.179125	0.769538	0.048*	
Cl1D	0.06670 (6)	0.51321 (4)	0.87121 (4)	0.03249 (12)	
Cl2D	0.23346 (6)	1.03530 (5)	0.36653 (3)	0.03456 (12)	
O1D	−0.07100 (19)	0.58552 (14)	0.63972 (10)	0.0312 (3)	
O2D	0.20954 (17)	0.84169 (14)	0.82467 (10)	0.0291 (3)	
H2OD	0.290786	0.797596	0.829106	0.044*	
N1D	0.1656 (2)	0.95880 (15)	0.54293 (11)	0.0273 (4)	
H1ND	0.175564	0.991871	0.493065	0.033*	
N2D	−0.06781 (19)	0.74273 (14)	0.89353 (10)	0.0229 (3)	
H2ND	−0.022048	0.667034	0.884151	0.028*	
C1D	0.1029 (2)	0.86763 (16)	0.56662 (12)	0.0228 (4)	
C2D	0.0544 (2)	0.83083 (18)	0.51153 (12)	0.0270 (4)	
H2D	0.063532	0.869664	0.458303	0.032*	
C3D	−0.0063 (2)	0.73890 (19)	0.53449 (13)	0.0268 (4)	
H3D	−0.041583	0.715036	0.497478	0.032*	
C4D	−0.0162 (2)	0.67966 (17)	0.61337 (12)	0.0235 (4)	
C5D	0.0319 (2)	0.71438 (16)	0.66832 (12)	0.0213 (4)	
H5D	0.025583	0.673017	0.720992	0.026*	
C6D	0.0904 (2)	0.81096 (16)	0.64657 (11)	0.0206 (3)	
C7D	0.1401 (2)	0.85548 (16)	0.69945 (12)	0.0209 (3)	
C8D	0.1991 (2)	0.94854 (17)	0.67111 (13)	0.0262 (4)	
H8D	0.231582	0.978738	0.705721	0.031*	
C9D	0.2114 (3)	0.99883 (18)	0.59143 (14)	0.0294 (4)	
H9D	0.253222	1.062607	0.572200	0.035*	
C10D	−0.1189 (3)	0.5456 (2)	0.58402 (15)	0.0324 (5)	
H10J	−0.200480	0.601346	0.561429	0.049*	
H10K	−0.150311	0.475584	0.609844	0.049*	
H10L	−0.038826	0.532567	0.542709	0.049*	
C11D	0.1265 (2)	0.80182 (16)	0.78614 (12)	0.0212 (4)	
H11D	0.160610	0.719065	0.794335	0.025*	
C12D	−0.0351 (2)	0.82913 (16)	0.82019 (11)	0.0201 (3)	
H12D	−0.094437	0.826679	0.780920	0.024*	
C13D	−0.0875 (2)	0.94454 (16)	0.83868 (12)	0.0255 (4)	
H13G	−0.143666	0.996950	0.797613	0.031*	
H13H	−0.002894	0.974518	0.840408	0.031*	
C14D	−0.1836 (2)	0.93303 (18)	0.91783 (12)	0.0261 (4)	
H14D	−0.228329	1.008771	0.927355	0.031*	
C15D	−0.0885 (3)	0.8651 (2)	0.98123 (14)	0.0345 (5)	
H15I	−0.017095	0.907207	0.984466	0.041*	
H15J	−0.149599	0.851443	1.032223	0.041*	
C16D	−0.0090 (3)	0.7541 (2)	0.96195 (13)	0.0310 (5)	
H16I	0.096581	0.751730	0.949199	0.037*	
H16J	−0.023979	0.691628	1.007349	0.037*	
C17D	−0.2290 (2)	0.75221 (19)	0.91170 (14)	0.0289 (4)	
H17I	−0.251475	0.697175	0.960561	0.035*	
H17J	−0.266155	0.736056	0.869327	0.035*	
C18D	−0.3037 (2)	0.8720 (2)	0.92047 (14)	0.0297 (4)	
H18D	−0.362888	0.866063	0.972839	0.036*	
C19D	−0.4040 (3)	0.9338 (2)	0.8595 (2)	0.0490 (7)	
H19D	−0.363443	0.945993	0.806359	0.059*	
C20D	−0.5429 (4)	0.9714 (4)	0.8761 (4)	0.0961 (19)	
H20I	−0.586299	0.960372	0.928753	0.115*	
H20J	−0.600859	1.009718	0.835423	0.115*	

### Atomic displacement parameters (Å^2^)

**Table:**

	*U*^11^	*U*^22^	*U*^33^	*U*^12^	*U*^13^	*U*^23^
Cl1A	0.0240 (2)	0.0471 (3)	0.0299 (3)	−0.0080 (2)	−0.00407 (19)	−0.0154 (2)
Cl2A	0.0678 (5)	0.0463 (4)	0.0365 (3)	0.0068 (3)	−0.0227 (3)	−0.0202 (3)
O1A	0.0425 (10)	0.0426 (10)	0.0321 (9)	−0.0248 (8)	−0.0057 (7)	0.0007 (7)
O2A	0.0345 (8)	0.0235 (7)	0.0377 (9)	−0.0080 (6)	0.0081 (7)	−0.0060 (7)
O3A	0.0631 (15)	0.0585 (14)	0.0380 (11)	−0.0099 (12)	−0.0171 (10)	−0.0007 (10)
N1A	0.0219 (8)	0.0533 (13)	0.0291 (9)	−0.0045 (8)	−0.0006 (7)	−0.0244 (9)
N2A	0.0235 (8)	0.0293 (9)	0.0213 (8)	−0.0108 (7)	0.0017 (6)	−0.0080 (7)
C1A	0.0172 (8)	0.0379 (11)	0.0240 (10)	−0.0023 (8)	−0.0003 (7)	−0.0138 (8)
C2A	0.0269 (10)	0.0415 (12)	0.0213 (10)	0.0003 (9)	−0.0029 (8)	−0.0070 (9)
C3A	0.0292 (11)	0.0352 (11)	0.0224 (10)	−0.0052 (9)	−0.0006 (8)	0.0005 (8)
C4A	0.0229 (9)	0.0316 (11)	0.0264 (10)	−0.0078 (8)	−0.0030 (8)	−0.0036 (8)
C5A	0.0198 (9)	0.0302 (10)	0.0210 (9)	−0.0070 (7)	−0.0011 (7)	−0.0058 (7)
C6A	0.0159 (8)	0.0308 (10)	0.0216 (9)	−0.0046 (7)	0.0003 (7)	−0.0102 (7)
C7A	0.0186 (8)	0.0310 (10)	0.0274 (10)	−0.0075 (7)	0.0044 (7)	−0.0133 (8)
C8A	0.0256 (10)	0.0433 (13)	0.0385 (13)	−0.0171 (10)	0.0117 (9)	−0.0227 (11)
C9A	0.0222 (10)	0.0524 (15)	0.0410 (13)	−0.0131 (10)	0.0059 (9)	−0.0295 (12)
C10A	0.0350 (12)	0.0464 (14)	0.0350 (12)	−0.0197 (11)	−0.0030 (10)	−0.0096 (11)
C11A	0.0225 (9)	0.0247 (9)	0.0252 (10)	−0.0075 (7)	0.0047 (7)	−0.0069 (7)
C12A	0.0223 (9)	0.0255 (9)	0.0168 (8)	−0.0083 (7)	0.0020 (7)	−0.0052 (7)
C13A	0.0232 (9)	0.0285 (10)	0.0220 (9)	−0.0084 (8)	0.0010 (7)	−0.0095 (7)
C14A	0.0218 (9)	0.0254 (9)	0.0240 (9)	−0.0084 (7)	0.0028 (7)	−0.0069 (7)
C15A	0.0279 (10)	0.0293 (10)	0.0259 (10)	−0.0113 (8)	0.0028 (8)	−0.0011 (8)
C16A	0.0301 (10)	0.0341 (11)	0.0211 (9)	−0.0105 (9)	−0.0007 (8)	−0.0042 (8)
C17A	0.0347 (11)	0.0280 (10)	0.0323 (11)	−0.0128 (9)	0.0051 (9)	−0.0149 (9)
C18A	0.0271 (10)	0.0268 (10)	0.0235 (9)	−0.0072 (8)	0.0038 (8)	−0.0098 (8)
C19A	0.0445 (13)	0.0271 (11)	0.0322 (12)	−0.0057 (10)	−0.0036 (10)	−0.0066 (9)
C20A	0.0450 (15)	0.0385 (14)	0.0548 (17)	0.0015 (11)	−0.0136 (13)	−0.0202 (13)
Cl1B	0.0217 (2)	0.0318 (3)	0.0343 (3)	−0.01018 (19)	−0.00112 (19)	−0.0025 (2)
Cl2B	0.0457 (3)	0.0364 (3)	0.0371 (3)	−0.0175 (3)	−0.0049 (3)	0.0000 (2)
O1B	0.0439 (10)	0.0494 (11)	0.0334 (9)	−0.0305 (9)	−0.0036 (8)	−0.0101 (8)
O2B	0.0277 (8)	0.0388 (9)	0.0420 (10)	−0.0061 (7)	−0.0019 (7)	−0.0254 (8)
N1B	0.0246 (8)	0.0247 (8)	0.0331 (10)	−0.0071 (7)	−0.0059 (7)	0.0004 (7)
N2B	0.0247 (8)	0.0365 (10)	0.0197 (8)	−0.0167 (7)	−0.0001 (6)	−0.0056 (7)
C1B	0.0168 (8)	0.0216 (9)	0.0309 (10)	−0.0030 (7)	−0.0035 (7)	−0.0052 (7)
C2B	0.0218 (9)	0.0291 (10)	0.0267 (10)	−0.0037 (8)	−0.0039 (8)	−0.0046 (8)
C3B	0.0235 (9)	0.0351 (11)	0.0299 (10)	−0.0079 (8)	−0.0050 (8)	−0.0105 (9)
C4B	0.0225 (9)	0.0305 (10)	0.0310 (11)	−0.0103 (8)	−0.0026 (8)	−0.0096 (8)
C5B	0.0205 (9)	0.0263 (9)	0.0285 (10)	−0.0087 (7)	−0.0014 (7)	−0.0068 (8)
C6B	0.0163 (8)	0.0213 (8)	0.0291 (10)	−0.0032 (7)	−0.0032 (7)	−0.0073 (7)
C7B	0.0166 (8)	0.0225 (9)	0.0312 (10)	−0.0023 (7)	−0.0034 (7)	−0.0085 (8)
C8B	0.0229 (9)	0.0253 (10)	0.0394 (12)	−0.0062 (8)	−0.0088 (8)	−0.0090 (9)
C9B	0.0262 (10)	0.0250 (10)	0.0446 (13)	−0.0100 (8)	−0.0083 (9)	−0.0035 (9)
C10B	0.085 (2)	0.067 (2)	0.0400 (15)	−0.059 (2)	−0.0094 (15)	−0.0007 (14)
C11B	0.0207 (9)	0.0311 (10)	0.0273 (10)	−0.0083 (8)	−0.0016 (7)	−0.0123 (8)
C12B	0.0215 (9)	0.0277 (9)	0.0200 (9)	−0.0110 (7)	−0.0007 (7)	−0.0072 (7)
C13B	0.0203 (9)	0.0277 (10)	0.0272 (10)	−0.0088 (7)	−0.0004 (7)	−0.0083 (8)
C14B	0.027 (4)	0.043 (4)	0.031 (3)	−0.021 (3)	−0.008 (3)	−0.004 (3)
C15B	0.036 (4)	0.057 (4)	0.017 (2)	−0.017 (3)	−0.007 (3)	−0.010 (3)
C16B	0.024 (3)	0.057 (5)	0.018 (4)	−0.027 (4)	0.000 (3)	−0.006 (3)
C17B	0.0378 (12)	0.0317 (11)	0.0288 (11)	−0.0136 (9)	−0.0067 (9)	0.0002 (9)
C18B	0.025 (3)	0.044 (4)	0.035 (4)	−0.011 (3)	−0.006 (2)	0.008 (3)
C19B	0.038 (3)	0.034 (2)	0.037 (3)	−0.010 (2)	−0.013 (2)	0.011 (2)
C20B	0.037 (3)	0.032 (3)	0.050 (3)	−0.005 (2)	−0.003 (3)	−0.005 (2)
C14E	0.022 (3)	0.050 (4)	0.022 (3)	−0.005 (3)	−0.005 (2)	−0.008 (3)
C15E	0.022 (3)	0.057 (5)	0.033 (3)	−0.005 (3)	−0.010 (2)	−0.018 (3)
C16E	0.029 (4)	0.050 (4)	0.016 (3)	0.001 (3)	−0.006 (3)	−0.014 (3)
C17E	0.0378 (12)	0.0317 (11)	0.0288 (11)	−0.0136 (9)	−0.0067 (9)	0.0002 (9)
C18E	0.034 (3)	0.032 (3)	0.028 (3)	−0.003 (2)	0.001 (2)	0.004 (2)
C19E	0.037 (3)	0.030 (2)	0.026 (3)	−0.004 (2)	−0.002 (2)	−0.005 (2)
C20E	0.032 (3)	0.048 (3)	0.051 (3)	−0.006 (2)	0.002 (3)	−0.014 (3)
Cl1C	0.0318 (3)	0.0249 (2)	0.0320 (3)	−0.00022 (19)	−0.0096 (2)	−0.01208 (19)
Cl2C	0.0380 (3)	0.0560 (4)	0.0234 (2)	−0.0067 (3)	−0.0067 (2)	−0.0154 (2)
O1C	0.0457 (10)	0.0315 (8)	0.0323 (9)	−0.0149 (8)	−0.0008 (8)	−0.0004 (7)
O2C	0.0212 (7)	0.0276 (7)	0.0276 (7)	−0.0055 (6)	0.0016 (6)	−0.0020 (6)
O3C	0.0751 (17)	0.0678 (16)	0.0339 (11)	−0.0225 (13)	−0.0060 (11)	−0.0120 (10)
N1C	0.0289 (9)	0.0384 (10)	0.0258 (9)	0.0041 (8)	−0.0096 (7)	−0.0147 (8)
N2C	0.0218 (8)	0.0198 (7)	0.0215 (8)	−0.0028 (6)	−0.0043 (6)	−0.0049 (6)
C1C	0.0252 (10)	0.0302 (10)	0.0218 (9)	0.0042 (8)	−0.0057 (8)	−0.0086 (8)
C2C	0.0363 (12)	0.0379 (12)	0.0207 (10)	0.0044 (10)	−0.0055 (9)	−0.0052 (9)
C3C	0.0372 (12)	0.0337 (12)	0.0223 (10)	−0.0005 (10)	0.0002 (9)	0.0014 (9)
C4C	0.0272 (10)	0.0251 (10)	0.0261 (10)	−0.0003 (8)	−0.0005 (8)	−0.0028 (8)
C5C	0.0232 (9)	0.0223 (9)	0.0203 (9)	−0.0009 (7)	−0.0025 (7)	−0.0036 (7)
C6C	0.0194 (8)	0.0247 (9)	0.0210 (9)	0.0025 (7)	−0.0027 (7)	−0.0074 (7)
C7C	0.0181 (8)	0.0224 (9)	0.0234 (9)	0.0013 (7)	−0.0033 (7)	−0.0077 (7)
C8C	0.0262 (10)	0.0275 (10)	0.0298 (11)	−0.0025 (8)	−0.0070 (8)	−0.0094 (8)
C9C	0.0300 (11)	0.0340 (12)	0.0363 (12)	0.0000 (9)	−0.0098 (9)	−0.0166 (10)
C10C	0.0416 (13)	0.0316 (12)	0.0404 (14)	−0.0139 (10)	−0.0063 (11)	−0.0042 (10)
C11C	0.0192 (8)	0.0213 (8)	0.0220 (9)	−0.0029 (7)	−0.0019 (7)	−0.0055 (7)
C12C	0.0190 (8)	0.0208 (8)	0.0182 (8)	−0.0030 (6)	−0.0026 (6)	−0.0035 (6)
C13C	0.0232 (9)	0.0218 (9)	0.0214 (9)	−0.0005 (7)	−0.0010 (7)	−0.0071 (7)
C14C	0.0263 (9)	0.0197 (9)	0.0236 (9)	0.0022 (7)	−0.0013 (8)	−0.0039 (7)
C15C	0.0335 (11)	0.0221 (9)	0.0221 (9)	−0.0022 (8)	−0.0004 (8)	−0.0017 (7)
C16C	0.0291 (10)	0.0254 (9)	0.0191 (9)	−0.0027 (8)	−0.0017 (7)	−0.0052 (7)
C17C	0.0209 (9)	0.0324 (11)	0.0297 (11)	−0.0047 (8)	−0.0059 (8)	−0.0063 (9)
C18C	0.0243 (9)	0.0304 (11)	0.0231 (9)	0.0026 (8)	−0.0036 (8)	−0.0053 (8)
C19C	0.0283 (11)	0.0437 (13)	0.0290 (11)	−0.0025 (10)	−0.0019 (9)	−0.0096 (10)
C20C	0.0278 (11)	0.0402 (13)	0.0480 (15)	0.0029 (10)	−0.0025 (10)	−0.0141 (11)
Cl1D	0.0256 (2)	0.0188 (2)	0.0475 (3)	−0.00282 (18)	0.0009 (2)	−0.0044 (2)
Cl2D	0.0342 (3)	0.0387 (3)	0.0260 (2)	−0.0122 (2)	0.0017 (2)	0.0003 (2)
O1D	0.0410 (9)	0.0312 (8)	0.0277 (8)	−0.0184 (7)	−0.0039 (7)	−0.0079 (6)
O2D	0.0253 (7)	0.0306 (8)	0.0353 (8)	−0.0050 (6)	−0.0128 (6)	−0.0093 (6)
N1D	0.0288 (9)	0.0226 (8)	0.0254 (9)	−0.0067 (7)	0.0034 (7)	−0.0010 (7)
N2D	0.0242 (8)	0.0186 (7)	0.0226 (8)	−0.0028 (6)	−0.0025 (6)	−0.0014 (6)
C1D	0.0222 (9)	0.0198 (8)	0.0224 (9)	−0.0039 (7)	0.0027 (7)	−0.0029 (7)
C2D	0.0295 (10)	0.0293 (10)	0.0182 (9)	−0.0054 (8)	−0.0003 (8)	−0.0021 (8)
C3D	0.0276 (10)	0.0299 (10)	0.0238 (10)	−0.0063 (8)	−0.0042 (8)	−0.0074 (8)
C4D	0.0238 (9)	0.0219 (9)	0.0253 (10)	−0.0058 (7)	0.0000 (7)	−0.0079 (7)
C5D	0.0227 (9)	0.0196 (8)	0.0204 (9)	−0.0050 (7)	−0.0009 (7)	−0.0038 (7)
C6D	0.0195 (8)	0.0187 (8)	0.0218 (9)	−0.0033 (7)	−0.0001 (7)	−0.0044 (7)
C7D	0.0187 (8)	0.0183 (8)	0.0233 (9)	−0.0022 (6)	−0.0003 (7)	−0.0045 (7)
C8D	0.0263 (10)	0.0222 (9)	0.0309 (11)	−0.0081 (8)	−0.0008 (8)	−0.0068 (8)
C9D	0.0313 (11)	0.0220 (9)	0.0333 (11)	−0.0109 (8)	0.0025 (9)	−0.0037 (8)
C10D	0.0351 (12)	0.0331 (11)	0.0364 (12)	−0.0130 (9)	−0.0052 (9)	−0.0145 (9)
C11D	0.0203 (8)	0.0193 (8)	0.0243 (9)	−0.0033 (7)	−0.0048 (7)	−0.0052 (7)
C12D	0.0219 (8)	0.0185 (8)	0.0182 (8)	−0.0022 (7)	−0.0042 (7)	−0.0026 (6)
C13D	0.0330 (10)	0.0160 (8)	0.0227 (9)	0.0000 (7)	−0.0026 (8)	−0.0023 (7)
C14D	0.0327 (11)	0.0232 (9)	0.0213 (9)	−0.0018 (8)	−0.0040 (8)	−0.0073 (7)
C15D	0.0450 (13)	0.0367 (12)	0.0239 (10)	−0.0074 (10)	−0.0122 (9)	−0.0069 (9)
C16D	0.0331 (11)	0.0330 (11)	0.0211 (10)	−0.0033 (9)	−0.0098 (8)	0.0036 (8)
C17D	0.0237 (10)	0.0274 (10)	0.0340 (11)	−0.0071 (8)	0.0008 (8)	−0.0060 (8)
C18D	0.0239 (10)	0.0316 (11)	0.0310 (11)	−0.0004 (8)	−0.0005 (8)	−0.0103 (9)
C19D	0.0428 (15)	0.0393 (14)	0.069 (2)	0.0064 (12)	−0.0291 (14)	−0.0193 (14)
C20D	0.053 (2)	0.089 (3)	0.167 (5)	0.037 (2)	−0.058 (3)	−0.082 (3)

### Geometric parameters (Å, º)

**Table:**

O1A—C4A	1.356 (3)	C16E—H16F	0.9900
O1A—C10A	1.434 (3)	C17E—C18E	1.508 (12)
O2A—C11A	1.412 (3)	C17E—H17E	0.9900
O2A—H2OA	0.8400	C17E—H17F	0.9900
O3A—H3E	0.89 (2)	C18E—C19E	1.525 (11)
O3A—H3F	0.86 (2)	C18E—H18E	1.0000
N1A—C9A	1.317 (4)	C19E—C20E	1.296 (9)
N1A—C1A	1.364 (3)	C19E—H19E	0.9500
N1A—H1NA	0.8800	C20E—H20E	0.9500
N2A—C17A	1.495 (3)	C20E—H20F	0.9500
N2A—C16A	1.500 (3)	O1C—C4C	1.354 (3)
N2A—C12A	1.517 (3)	O1C—C10C	1.437 (3)
N2A—H2NA	1.0000	O2C—C11C	1.407 (2)
C1A—C2A	1.407 (3)	O2C—H2OC	0.8400
C1A—C6A	1.412 (3)	O3C—H3G	0.88 (3)
C2A—C3A	1.353 (4)	O3C—H3H	0.89 (3)
C2A—H2A	0.9500	N1C—C9C	1.318 (3)
C3A—C4A	1.422 (3)	N1C—C1C	1.367 (3)
C3A—H3A	0.9500	N1C—H1NC	0.8800
C4A—C5A	1.377 (3)	N2C—C17C	1.505 (3)
C5A—C6A	1.426 (3)	N2C—C16C	1.506 (3)
C5A—H5A	0.9500	N2C—C12C	1.511 (3)
C6A—C7A	1.426 (3)	N2C—H2NC	1.0000
C7A—C8A	1.382 (3)	C1C—C2C	1.409 (3)
C7A—C11A	1.519 (3)	C1C—C6C	1.424 (3)
C8A—C9A	1.387 (4)	C2C—C3C	1.359 (4)
C8A—H8A	0.9500	C2C—H2C	0.9500
C9A—H9A	0.9500	C3C—C4C	1.421 (3)
C10A—H10A	0.9800	C3C—H3C	0.9500
C10A—H10B	0.9800	C4C—C5C	1.385 (3)
C10A—H10C	0.9800	C5C—C6C	1.425 (3)
C11A—C12A	1.540 (3)	C5C—H5C	0.9500
C11A—H11A	1.0000	C6C—C7C	1.423 (3)
C12A—C13A	1.542 (3)	C7C—C8C	1.383 (3)
C12A—H12A	1.0000	C7C—C11C	1.521 (3)
C13A—C14A	1.535 (3)	C8C—C9C	1.394 (3)
C13A—H13A	0.9900	C8C—H8C	0.9500
C13A—H13B	0.9900	C9C—H9C	0.9500
C14A—C15A	1.524 (3)	C10C—H10G	0.9800
C14A—C18A	1.543 (3)	C10C—H10H	0.9800
C14A—H14A	1.0000	C10C—H10I	0.9800
C15A—C16A	1.530 (3)	C11C—C12C	1.539 (3)
C15A—H15A	0.9900	C11C—H11C	1.0000
C15A—H15B	0.9900	C12C—C13C	1.545 (3)
C16A—H16A	0.9900	C12C—H12C	1.0000
C16A—H16B	0.9900	C13C—C14C	1.536 (3)
C17A—C18A	1.546 (3)	C13C—H13E	0.9900
C17A—H17A	0.9900	C13C—H13F	0.9900
C17A—H17B	0.9900	C14C—C15C	1.531 (3)
C18A—C19A	1.508 (3)	C14C—C18C	1.546 (3)
C18A—H18A	1.0000	C14C—H14C	1.0000
C19A—C20A	1.315 (4)	C15C—C16C	1.533 (3)
C19A—H19A	0.9500	C15C—H15G	0.9900
C20A—H20A	0.9500	C15C—H15H	0.9900
C20A—H20B	0.9500	C16C—H16G	0.9900
O1B—C4B	1.357 (3)	C16C—H16H	0.9900
O1B—C10B	1.427 (3)	C17C—C18C	1.548 (3)
O2B—C11B	1.413 (3)	C17C—H17G	0.9900
O2B—H2OB	0.8400	C17C—H17H	0.9900
N1B—C9B	1.326 (3)	C18C—C19C	1.496 (3)
N1B—C1B	1.367 (3)	C18C—H18C	1.0000
N1B—H1NB	0.8800	C19C—C20C	1.311 (4)
N2B—C16B	1.467 (16)	C19C—H19C	0.9500
N2B—C17E	1.495 (3)	C20C—H20G	0.9500
N2B—C17B	1.495 (3)	C20C—H20H	0.9500
N2B—C12B	1.511 (3)	O1D—C4D	1.363 (2)
N2B—C16E	1.558 (16)	O1D—C10D	1.434 (3)
N2B—H2NB	1.0000	O2D—C11D	1.409 (2)
C1B—C2B	1.413 (3)	O2D—H2OD	0.8400
C1B—C6B	1.416 (3)	N1D—C9D	1.313 (3)
C2B—C3B	1.358 (3)	N1D—C1D	1.373 (3)
C2B—H2B	0.9500	N1D—H1ND	0.8800
C3B—C4B	1.415 (3)	N2D—C17D	1.500 (3)
C3B—H3B	0.9500	N2D—C16D	1.505 (3)
C4B—C5B	1.378 (3)	N2D—C12D	1.510 (3)
C5B—C6B	1.425 (3)	N2D—H2ND	1.0000
C5B—H5B	0.9500	C1D—C2D	1.403 (3)
C6B—C7B	1.429 (3)	C1D—C6D	1.421 (3)
C7B—C8B	1.386 (3)	C2D—C3D	1.370 (3)
C7B—C11B	1.525 (3)	C2D—H2D	0.9500
C8B—C9B	1.388 (3)	C3D—C4D	1.413 (3)
C8B—H8B	0.9500	C3D—H3D	0.9500
C9B—H9B	0.9500	C4D—C5D	1.382 (3)
C10B—H10D	0.9800	C5D—C6D	1.411 (3)
C10B—H10E	0.9800	C5D—H5D	0.9500
C10B—H10F	0.9800	C6D—C7D	1.438 (3)
C11B—C12B	1.542 (3)	C7D—C8D	1.375 (3)
C11B—H11B	1.0000	C7D—C11D	1.519 (3)
C12B—C13B	1.538 (3)	C8D—C9D	1.399 (3)
C12B—H12B	1.0000	C8D—H8D	0.9500
C13B—C14B	1.513 (12)	C9D—H9D	0.9500
C13B—C14E	1.557 (12)	C10D—H10J	0.9800
C13B—H13C	0.9900	C10D—H10K	0.9800
C13B—H13D	0.9900	C10D—H10L	0.9800
C14B—C18B	1.533 (12)	C11D—C12D	1.545 (3)
C14B—C15B	1.533 (12)	C11D—H11D	1.0000
C14B—H14B	1.0000	C12D—C13D	1.537 (3)
C15B—C16B	1.537 (12)	C12D—H12D	1.0000
C15B—H15C	0.9900	C13D—C14D	1.538 (3)
C15B—H15D	0.9900	C13D—H13G	0.9900
C16B—H16C	0.9900	C13D—H13H	0.9900
C16B—H16D	0.9900	C14D—C15D	1.528 (3)
C17B—C18B	1.592 (11)	C14D—C18D	1.535 (3)
C17B—H17C	0.9900	C14D—H14D	1.0000
C17B—H17D	0.9900	C15D—C16D	1.527 (4)
C18B—C19B	1.529 (11)	C15D—H15I	0.9900
C18B—H18B	1.0000	C15D—H15J	0.9900
C19B—C20B	1.307 (10)	C16D—H16I	0.9900
C19B—H19B	0.9500	C16D—H16J	0.9900
C20B—H20C	0.9500	C17D—C18D	1.551 (3)
C20B—H20D	0.9500	C17D—H17I	0.9900
C14E—C18E	1.526 (13)	C17D—H17J	0.9900
C14E—C15E	1.530 (12)	C18D—C19D	1.513 (4)
C14E—H14E	1.0000	C18D—H18D	1.0000
C15E—C16E	1.509 (12)	C19D—C20D	1.304 (5)
C15E—H15E	0.9900	C19D—H19D	0.9500
C15E—H15F	0.9900	C20D—H20I	0.9500
C16E—H16E	0.9900	C20D—H20J	0.9500
			
C4A—O1A—C10A	118.64 (18)	N2B—C16E—H16F	109.8
C11A—O2A—H2OA	109.5	H16E—C16E—H16F	108.2
H3E—O3A—H3F	110 (4)	N2B—C17E—C18E	113.4 (5)
C9A—N1A—C1A	123.2 (2)	N2B—C17E—H17E	108.9
C9A—N1A—H1NA	118.4	C18E—C17E—H17E	108.9
C1A—N1A—H1NA	118.4	N2B—C17E—H17F	108.9
C17A—N2A—C16A	109.24 (17)	C18E—C17E—H17F	108.9
C17A—N2A—C12A	108.74 (17)	H17E—C17E—H17F	107.7
C16A—N2A—C12A	113.34 (16)	C17E—C18E—C19E	110.0 (8)
C17A—N2A—H2NA	108.5	C17E—C18E—C14E	106.6 (9)
C16A—N2A—H2NA	108.5	C19E—C18E—C14E	113.1 (9)
C12A—N2A—H2NA	108.5	C17E—C18E—H18E	109.0
N1A—C1A—C2A	119.5 (2)	C19E—C18E—H18E	109.0
N1A—C1A—C6A	119.5 (2)	C14E—C18E—H18E	109.0
C2A—C1A—C6A	121.0 (2)	C20E—C19E—C18E	124.5 (8)
C3A—C2A—C1A	119.8 (2)	C20E—C19E—H19E	117.8
C3A—C2A—H2A	120.1	C18E—C19E—H19E	117.8
C1A—C2A—H2A	120.1	C19E—C20E—H20E	120.0
C2A—C3A—C4A	120.4 (2)	C19E—C20E—H20F	120.0
C2A—C3A—H3A	119.8	H20E—C20E—H20F	120.0
C4A—C3A—H3A	119.8	C4C—O1C—C10C	118.12 (18)
O1A—C4A—C5A	125.2 (2)	C11C—O2C—H2OC	109.5
O1A—C4A—C3A	113.8 (2)	H3G—O3C—H3H	109 (4)
C5A—C4A—C3A	121.1 (2)	C9C—N1C—C1C	123.2 (2)
C4A—C5A—C6A	119.2 (2)	C9C—N1C—H1NC	118.4
C4A—C5A—H5A	120.4	C1C—N1C—H1NC	118.4
C6A—C5A—H5A	120.4	C17C—N2C—C16C	108.82 (16)
C1A—C6A—C5A	118.52 (19)	C17C—N2C—C12C	108.80 (16)
C1A—C6A—C7A	117.45 (19)	C16C—N2C—C12C	113.89 (16)
C5A—C6A—C7A	124.02 (19)	C17C—N2C—H2NC	108.4
C8A—C7A—C6A	119.4 (2)	C16C—N2C—H2NC	108.4
C8A—C7A—C11A	118.4 (2)	C12C—N2C—H2NC	108.4
C6A—C7A—C11A	122.18 (18)	N1C—C1C—C2C	120.0 (2)
C7A—C8A—C9A	120.3 (2)	N1C—C1C—C6C	119.3 (2)
C7A—C8A—H8A	119.8	C2C—C1C—C6C	120.7 (2)
C9A—C8A—H8A	119.8	C3C—C2C—C1C	120.4 (2)
N1A—C9A—C8A	120.0 (2)	C3C—C2C—H2C	119.8
N1A—C9A—H9A	120.0	C1C—C2C—H2C	119.8
C8A—C9A—H9A	120.0	C2C—C3C—C4C	120.1 (2)
O1A—C10A—H10A	109.5	C2C—C3C—H3C	120.0
O1A—C10A—H10B	109.5	C4C—C3C—H3C	120.0
H10A—C10A—H10B	109.5	O1C—C4C—C5C	125.2 (2)
O1A—C10A—H10C	109.5	O1C—C4C—C3C	114.0 (2)
H10A—C10A—H10C	109.5	C5C—C4C—C3C	120.8 (2)
H10B—C10A—H10C	109.5	C4C—C5C—C6C	120.0 (2)
O2A—C11A—C7A	112.26 (18)	C4C—C5C—H5C	120.0
O2A—C11A—C12A	108.25 (16)	C6C—C5C—H5C	120.0
C7A—C11A—C12A	107.15 (17)	C7C—C6C—C1C	117.37 (19)
O2A—C11A—H11A	109.7	C7C—C6C—C5C	124.74 (18)
C7A—C11A—H11A	109.7	C1C—C6C—C5C	117.89 (19)
C12A—C11A—H11A	109.7	C8C—C7C—C6C	119.77 (19)
N2A—C12A—C11A	110.88 (17)	C8C—C7C—C11C	118.59 (18)
N2A—C12A—C13A	108.55 (15)	C6C—C7C—C11C	121.64 (18)
C11A—C12A—C13A	113.99 (17)	C7C—C8C—C9C	120.2 (2)
N2A—C12A—H12A	107.7	C7C—C8C—H8C	119.9
C11A—C12A—H12A	107.7	C9C—C8C—H8C	119.9
C13A—C12A—H12A	107.7	N1C—C9C—C8C	120.1 (2)
C14A—C13A—C12A	109.42 (17)	N1C—C9C—H9C	120.0
C14A—C13A—H13A	109.8	C8C—C9C—H9C	120.0
C12A—C13A—H13A	109.8	O1C—C10C—H10G	109.5
C14A—C13A—H13B	109.8	O1C—C10C—H10H	109.5
C12A—C13A—H13B	109.8	H10G—C10C—H10H	109.5
H13A—C13A—H13B	108.2	O1C—C10C—H10I	109.5
C15A—C14A—C13A	109.65 (17)	H10G—C10C—H10I	109.5
C15A—C14A—C18A	108.24 (18)	H10H—C10C—H10I	109.5
C13A—C14A—C18A	109.56 (16)	O2C—C11C—C7C	111.02 (16)
C15A—C14A—H14A	109.8	O2C—C11C—C12C	109.11 (16)
C13A—C14A—H14A	109.8	C7C—C11C—C12C	110.27 (16)
C18A—C14A—H14A	109.8	O2C—C11C—H11C	108.8
C14A—C15A—C16A	110.26 (17)	C7C—C11C—H11C	108.8
C14A—C15A—H15A	109.6	C12C—C11C—H11C	108.8
C16A—C15A—H15A	109.6	N2C—C12C—C11C	110.77 (15)
C14A—C15A—H15B	109.6	N2C—C12C—C13C	107.78 (15)
C16A—C15A—H15B	109.6	C11C—C12C—C13C	115.65 (16)
H15A—C15A—H15B	108.1	N2C—C12C—H12C	107.4
N2A—C16A—C15A	108.48 (18)	C11C—C12C—H12C	107.4
N2A—C16A—H16A	110.0	C13C—C12C—H12C	107.4
C15A—C16A—H16A	110.0	C14C—C13C—C12C	109.34 (16)
N2A—C16A—H16B	110.0	C14C—C13C—H13E	109.8
C15A—C16A—H16B	110.0	C12C—C13C—H13E	109.8
H16A—C16A—H16B	108.4	C14C—C13C—H13F	109.8
N2A—C17A—C18A	110.19 (17)	C12C—C13C—H13F	109.8
N2A—C17A—H17A	109.6	H13E—C13C—H13F	108.3
C18A—C17A—H17A	109.6	C15C—C14C—C13C	108.97 (17)
N2A—C17A—H17B	109.6	C15C—C14C—C18C	107.20 (18)
C18A—C17A—H17B	109.6	C13C—C14C—C18C	111.80 (18)
H17A—C17A—H17B	108.1	C15C—C14C—H14C	109.6
C19A—C18A—C14A	112.10 (19)	C13C—C14C—H14C	109.6
C19A—C18A—C17A	112.50 (19)	C18C—C14C—H14C	109.6
C14A—C18A—C17A	107.75 (17)	C14C—C15C—C16C	108.28 (17)
C19A—C18A—H18A	108.1	C14C—C15C—H15G	110.0
C14A—C18A—H18A	108.1	C16C—C15C—H15G	110.0
C17A—C18A—H18A	108.1	C14C—C15C—H15H	110.0
C20A—C19A—C18A	123.7 (3)	C16C—C15C—H15H	110.0
C20A—C19A—H19A	118.2	H15G—C15C—H15H	108.4
C18A—C19A—H19A	118.2	N2C—C16C—C15C	108.71 (16)
C19A—C20A—H20A	120.0	N2C—C16C—H16G	109.9
C19A—C20A—H20B	120.0	C15C—C16C—H16G	109.9
H20A—C20A—H20B	120.0	N2C—C16C—H16H	109.9
C4B—O1B—C10B	117.6 (2)	C15C—C16C—H16H	109.9
C11B—O2B—H2OB	109.5	H16G—C16C—H16H	108.3
C9B—N1B—C1B	122.5 (2)	N2C—C17C—C18C	108.78 (17)
C9B—N1B—H1NB	118.8	N2C—C17C—H17G	109.9
C1B—N1B—H1NB	118.8	C18C—C17C—H17G	109.9
C16B—N2B—C17B	113.4 (7)	N2C—C17C—H17H	109.9
C16B—N2B—C12B	112.7 (5)	C18C—C17C—H17H	109.9
C17E—N2B—C12B	108.50 (16)	H17G—C17C—H17H	108.3
C17B—N2B—C12B	108.50 (16)	C19C—C18C—C14C	113.79 (19)
C17E—N2B—C16E	105.9 (6)	C19C—C18C—C17C	112.0 (2)
C12B—N2B—C16E	113.6 (6)	C14C—C18C—C17C	107.21 (17)
C16B—N2B—H2NB	107.3	C19C—C18C—H18C	107.9
C17B—N2B—H2NB	107.3	C14C—C18C—H18C	107.9
C12B—N2B—H2NB	107.3	C17C—C18C—H18C	107.9
N1B—C1B—C2B	118.6 (2)	C20C—C19C—C18C	125.4 (2)
N1B—C1B—C6B	119.98 (19)	C20C—C19C—H19C	117.3
C2B—C1B—C6B	121.41 (18)	C18C—C19C—H19C	117.3
C3B—C2B—C1B	119.0 (2)	C19C—C20C—H20G	120.0
C3B—C2B—H2B	120.5	C19C—C20C—H20H	120.0
C1B—C2B—H2B	120.5	H20G—C20C—H20H	120.0
C2B—C3B—C4B	120.9 (2)	C4D—O1D—C10D	117.75 (18)
C2B—C3B—H3B	119.6	C11D—O2D—H2OD	109.5
C4B—C3B—H3B	119.6	C9D—N1D—C1D	122.85 (19)
O1B—C4B—C5B	125.8 (2)	C9D—N1D—H1ND	118.6
O1B—C4B—C3B	112.9 (2)	C1D—N1D—H1ND	118.6
C5B—C4B—C3B	121.2 (2)	C17D—N2D—C16D	109.72 (17)
C4B—C5B—C6B	119.3 (2)	C17D—N2D—C12D	109.35 (16)
C4B—C5B—H5B	120.3	C16D—N2D—C12D	112.51 (16)
C6B—C5B—H5B	120.3	C17D—N2D—H2ND	108.4
C1B—C6B—C5B	118.15 (19)	C16D—N2D—H2ND	108.4
C1B—C6B—C7B	117.61 (18)	C12D—N2D—H2ND	108.4
C5B—C6B—C7B	124.2 (2)	N1D—C1D—C2D	119.60 (19)
C8B—C7B—C6B	119.0 (2)	N1D—C1D—C6D	119.32 (19)
C8B—C7B—C11B	119.17 (19)	C2D—C1D—C6D	121.07 (19)
C6B—C7B—C11B	121.84 (18)	C3D—C2D—C1D	119.99 (19)
C7B—C8B—C9B	120.7 (2)	C3D—C2D—H2D	120.0
C7B—C8B—H8B	119.7	C1D—C2D—H2D	120.0
C9B—C8B—H8B	119.7	C2D—C3D—C4D	119.8 (2)
N1B—C9B—C8B	120.2 (2)	C2D—C3D—H3D	120.1
N1B—C9B—H9B	119.9	C4D—C3D—H3D	120.1
C8B—C9B—H9B	119.9	O1D—C4D—C5D	116.21 (19)
O1B—C10B—H10D	109.5	O1D—C4D—C3D	122.86 (19)
O1B—C10B—H10E	109.5	C5D—C4D—C3D	120.92 (19)
H10D—C10B—H10E	109.5	C4D—C5D—C6D	120.33 (18)
O1B—C10B—H10F	109.5	C4D—C5D—H5D	119.8
H10D—C10B—H10F	109.5	C6D—C5D—H5D	119.8
H10E—C10B—H10F	109.5	C5D—C6D—C1D	117.82 (18)
O2B—C11B—C7B	111.21 (18)	C5D—C6D—C7D	124.64 (18)
O2B—C11B—C12B	108.71 (17)	C1D—C6D—C7D	117.53 (18)
C7B—C11B—C12B	108.69 (16)	C8D—C7D—C6D	119.44 (19)
O2B—C11B—H11B	109.4	C8D—C7D—C11D	119.73 (18)
C7B—C11B—H11B	109.4	C6D—C7D—C11D	120.82 (17)
C12B—C11B—H11B	109.4	C7D—C8D—C9D	120.1 (2)
N2B—C12B—C13B	107.80 (16)	C7D—C8D—H8D	119.9
N2B—C12B—C11B	111.84 (16)	C9D—C8D—H8D	119.9
C13B—C12B—C11B	113.91 (16)	N1D—C9D—C8D	120.7 (2)
N2B—C12B—H12B	107.7	N1D—C9D—H9D	119.7
C13B—C12B—H12B	107.7	C8D—C9D—H9D	119.7
C11B—C12B—H12B	107.7	O1D—C10D—H10J	109.5
C14B—C13B—C12B	109.5 (5)	O1D—C10D—H10K	109.5
C12B—C13B—C14E	110.2 (6)	H10J—C10D—H10K	109.5
C14B—C13B—H13C	109.8	O1D—C10D—H10L	109.5
C12B—C13B—H13C	109.8	H10J—C10D—H10L	109.5
C14B—C13B—H13D	109.8	H10K—C10D—H10L	109.5
C12B—C13B—H13D	109.8	O2D—C11D—C7D	111.48 (17)
H13C—C13B—H13D	108.2	O2D—C11D—C12D	109.31 (16)
C13B—C14B—C18B	108.8 (10)	C7D—C11D—C12D	108.49 (15)
C13B—C14B—C15B	108.5 (9)	O2D—C11D—H11D	109.2
C18B—C14B—C15B	110.0 (8)	C7D—C11D—H11D	109.2
C13B—C14B—H14B	109.8	C12D—C11D—H11D	109.2
C18B—C14B—H14B	109.8	N2D—C12D—C13D	107.84 (16)
C15B—C14B—H14B	109.8	N2D—C12D—C11D	111.11 (15)
C14B—C15B—C16B	108.8 (9)	C13D—C12D—C11D	114.79 (17)
C14B—C15B—H15C	109.9	N2D—C12D—H12D	107.6
C16B—C15B—H15C	109.9	C13D—C12D—H12D	107.6
C14B—C15B—H15D	109.9	C11D—C12D—H12D	107.6
C16B—C15B—H15D	109.9	C12D—C13D—C14D	108.96 (16)
H15C—C15B—H15D	108.3	C12D—C13D—H13G	109.9
N2B—C16B—C15B	108.5 (8)	C14D—C13D—H13G	109.9
N2B—C16B—H16C	110.0	C12D—C13D—H13H	109.9
C15B—C16B—H16C	110.0	C14D—C13D—H13H	109.9
N2B—C16B—H16D	110.0	H13G—C13D—H13H	108.3
C15B—C16B—H16D	110.0	C15D—C14D—C18D	108.56 (19)
H16C—C16B—H16D	108.4	C15D—C14D—C13D	108.52 (19)
N2B—C17B—C18B	105.9 (5)	C18D—C14D—C13D	110.97 (18)
N2B—C17B—H17C	110.6	C15D—C14D—H14D	109.6
C18B—C17B—H17C	110.6	C18D—C14D—H14D	109.6
N2B—C17B—H17D	110.6	C13D—C14D—H14D	109.6
C18B—C17B—H17D	110.6	C16D—C15D—C14D	109.23 (18)
H17C—C17B—H17D	108.7	C16D—C15D—H15I	109.8
C19B—C18B—C14B	110.3 (9)	C14D—C15D—H15I	109.8
C19B—C18B—C17B	117.4 (8)	C16D—C15D—H15J	109.8
C14B—C18B—C17B	108.9 (9)	C14D—C15D—H15J	109.8
C19B—C18B—H18B	106.6	H15I—C15D—H15J	108.3
C14B—C18B—H18B	106.6	N2D—C16D—C15D	108.66 (17)
C17B—C18B—H18B	106.6	N2D—C16D—H16I	110.0
C20B—C19B—C18B	126.8 (7)	C15D—C16D—H16I	110.0
C20B—C19B—H19B	116.6	N2D—C16D—H16J	110.0
C18B—C19B—H19B	116.6	C15D—C16D—H16J	110.0
C19B—C20B—H20C	120.0	H16I—C16D—H16J	108.3
C19B—C20B—H20D	120.0	N2D—C17D—C18D	109.40 (18)
H20C—C20B—H20D	120.0	N2D—C17D—H17I	109.8
C18E—C14E—C15E	109.9 (9)	C18D—C17D—H17I	109.8
C18E—C14E—C13B	110.2 (10)	N2D—C17D—H17J	109.8
C15E—C14E—C13B	108.5 (9)	C18D—C17D—H17J	109.8
C18E—C14E—H14E	109.4	H17I—C17D—H17J	108.2
C15E—C14E—H14E	109.4	C19D—C18D—C14D	112.2 (2)
C13B—C14E—H14E	109.4	C19D—C18D—C17D	112.4 (2)
C16E—C15E—C14E	109.4 (10)	C14D—C18D—C17D	107.59 (17)
C16E—C15E—H15E	109.8	C19D—C18D—H18D	108.2
C14E—C15E—H15E	109.8	C14D—C18D—H18D	108.2
C16E—C15E—H15F	109.8	C17D—C18D—H18D	108.2
C14E—C15E—H15F	109.8	C20D—C19D—C18D	123.5 (4)
H15E—C15E—H15F	108.2	C20D—C19D—H19D	118.2
C15E—C16E—N2B	109.5 (9)	C18D—C19D—H19D	118.2
C15E—C16E—H16E	109.8	C19D—C20D—H20I	120.0
N2B—C16E—H16E	109.8	C19D—C20D—H20J	120.0
C15E—C16E—H16F	109.8	H20I—C20D—H20J	120.0
			
C9A—N1A—C1A—C2A	−178.1 (2)	C12B—N2B—C16E—C15E	56.9 (11)
C9A—N1A—C1A—C6A	1.3 (3)	C12B—N2B—C17E—C18E	−62.2 (6)
N1A—C1A—C2A—C3A	177.5 (2)	C16E—N2B—C17E—C18E	60.1 (8)
C6A—C1A—C2A—C3A	−2.0 (3)	N2B—C17E—C18E—C19E	123.2 (7)
C1A—C2A—C3A—C4A	−0.2 (3)	N2B—C17E—C18E—C14E	0.3 (10)
C10A—O1A—C4A—C5A	−4.8 (4)	C15E—C14E—C18E—C17E	−61.1 (11)
C10A—O1A—C4A—C3A	174.7 (2)	C13B—C14E—C18E—C17E	58.4 (10)
C2A—C3A—C4A—O1A	−177.3 (2)	C15E—C14E—C18E—C19E	178.0 (9)
C2A—C3A—C4A—C5A	2.3 (4)	C13B—C14E—C18E—C19E	−62.5 (12)
O1A—C4A—C5A—C6A	177.5 (2)	C17E—C18E—C19E—C20E	132.2 (8)
C3A—C4A—C5A—C6A	−2.0 (3)	C14E—C18E—C19E—C20E	−108.8 (11)
N1A—C1A—C6A—C5A	−177.24 (19)	C9C—N1C—C1C—C2C	177.2 (2)
C2A—C1A—C6A—C5A	2.2 (3)	C9C—N1C—C1C—C6C	−2.5 (3)
N1A—C1A—C6A—C7A	1.9 (3)	N1C—C1C—C2C—C3C	178.6 (2)
C2A—C1A—C6A—C7A	−178.7 (2)	C6C—C1C—C2C—C3C	−1.6 (3)
C4A—C5A—C6A—C1A	−0.2 (3)	C1C—C2C—C3C—C4C	−1.4 (4)
C4A—C5A—C6A—C7A	−179.2 (2)	C10C—O1C—C4C—C5C	−2.0 (3)
C1A—C6A—C7A—C8A	−4.1 (3)	C10C—O1C—C4C—C3C	178.3 (2)
C5A—C6A—C7A—C8A	175.0 (2)	C2C—C3C—C4C—O1C	−178.5 (2)
C1A—C6A—C7A—C11A	173.52 (19)	C2C—C3C—C4C—C5C	1.8 (3)
C5A—C6A—C7A—C11A	−7.4 (3)	O1C—C4C—C5C—C6C	−178.8 (2)
C6A—C7A—C8A—C9A	3.3 (3)	C3C—C4C—C5C—C6C	0.8 (3)
C11A—C7A—C8A—C9A	−174.4 (2)	N1C—C1C—C6C—C7C	3.9 (3)
C1A—N1A—C9A—C8A	−2.2 (4)	C2C—C1C—C6C—C7C	−175.83 (19)
C7A—C8A—C9A—N1A	−0.1 (4)	N1C—C1C—C6C—C5C	−176.10 (18)
C8A—C7A—C11A—O2A	−15.4 (3)	C2C—C1C—C6C—C5C	4.2 (3)
C6A—C7A—C11A—O2A	166.95 (19)	C4C—C5C—C6C—C7C	176.27 (19)
C8A—C7A—C11A—C12A	103.3 (2)	C4C—C5C—C6C—C1C	−3.7 (3)
C6A—C7A—C11A—C12A	−74.3 (2)	C1C—C6C—C7C—C8C	−2.3 (3)
C17A—N2A—C12A—C11A	−171.57 (16)	C5C—C6C—C7C—C8C	177.67 (19)
C16A—N2A—C12A—C11A	66.7 (2)	C1C—C6C—C7C—C11C	178.10 (18)
C17A—N2A—C12A—C13A	62.5 (2)	C5C—C6C—C7C—C11C	−1.9 (3)
C16A—N2A—C12A—C13A	−59.2 (2)	C6C—C7C—C8C—C9C	−0.7 (3)
O2A—C11A—C12A—N2A	−79.9 (2)	C11C—C7C—C8C—C9C	178.89 (19)
C7A—C11A—C12A—N2A	158.77 (16)	C1C—N1C—C9C—C8C	−0.6 (3)
O2A—C11A—C12A—C13A	42.9 (2)	C7C—C8C—C9C—N1C	2.3 (3)
C7A—C11A—C12A—C13A	−78.4 (2)	C8C—C7C—C11C—O2C	−11.0 (2)
N2A—C12A—C13A—C14A	−0.8 (2)	C6C—C7C—C11C—O2C	168.60 (17)
C11A—C12A—C13A—C14A	−124.92 (18)	C8C—C7C—C11C—C12C	110.1 (2)
C12A—C13A—C14A—C15A	59.1 (2)	C6C—C7C—C11C—C12C	−70.3 (2)
C12A—C13A—C14A—C18A	−59.6 (2)	C17C—N2C—C12C—C11C	−174.33 (16)
C13A—C14A—C15A—C16A	−61.0 (2)	C16C—N2C—C12C—C11C	64.1 (2)
C18A—C14A—C15A—C16A	58.5 (2)	C17C—N2C—C12C—C13C	58.2 (2)
C17A—N2A—C16A—C15A	−63.6 (2)	C16C—N2C—C12C—C13C	−63.3 (2)
C12A—N2A—C16A—C15A	57.8 (2)	O2C—C11C—C12C—N2C	−77.72 (19)
C14A—C15A—C16A—N2A	3.2 (2)	C7C—C11C—C12C—N2C	160.10 (15)
C16A—N2A—C17A—C18A	60.3 (2)	O2C—C11C—C12C—C13C	45.3 (2)
C12A—N2A—C17A—C18A	−63.8 (2)	C7C—C11C—C12C—C13C	−76.9 (2)
C15A—C14A—C18A—C19A	174.51 (18)	N2C—C12C—C13C—C14C	7.0 (2)
C13A—C14A—C18A—C19A	−66.0 (2)	C11C—C12C—C13C—C14C	−117.55 (19)
C15A—C14A—C18A—C17A	−61.2 (2)	C12C—C13C—C14C—C15C	55.6 (2)
C13A—C14A—C18A—C17A	58.4 (2)	C12C—C13C—C14C—C18C	−62.7 (2)
N2A—C17A—C18A—C19A	126.7 (2)	C13C—C14C—C15C—C16C	−68.2 (2)
N2A—C17A—C18A—C14A	2.6 (2)	C18C—C14C—C15C—C16C	53.0 (2)
C14A—C18A—C19A—C20A	−89.7 (3)	C17C—N2C—C16C—C15C	−70.3 (2)
C17A—C18A—C19A—C20A	148.7 (3)	C12C—N2C—C16C—C15C	51.2 (2)
C9B—N1B—C1B—C2B	−177.6 (2)	C14C—C15C—C16C—N2C	14.0 (2)
C9B—N1B—C1B—C6B	1.4 (3)	C16C—N2C—C17C—C18C	52.0 (2)
N1B—C1B—C2B—C3B	−179.0 (2)	C12C—N2C—C17C—C18C	−72.6 (2)
C6B—C1B—C2B—C3B	2.1 (3)	C15C—C14C—C18C—C19C	165.20 (18)
C1B—C2B—C3B—C4B	0.4 (3)	C13C—C14C—C18C—C19C	−75.4 (2)
C10B—O1B—C4B—C5B	6.3 (4)	C15C—C14C—C18C—C17C	−70.4 (2)
C10B—O1B—C4B—C3B	−176.2 (3)	C13C—C14C—C18C—C17C	48.9 (2)
C2B—C3B—C4B—O1B	179.5 (2)	N2C—C17C—C18C—C19C	141.08 (19)
C2B—C3B—C4B—C5B	−2.8 (3)	N2C—C17C—C18C—C14C	15.6 (2)
O1B—C4B—C5B—C6B	−180.0 (2)	C14C—C18C—C19C—C20C	−114.1 (3)
C3B—C4B—C5B—C6B	2.7 (3)	C17C—C18C—C19C—C20C	124.2 (3)
N1B—C1B—C6B—C5B	178.96 (19)	C9D—N1D—C1D—C2D	178.9 (2)
C2B—C1B—C6B—C5B	−2.1 (3)	C9D—N1D—C1D—C6D	−2.1 (3)
N1B—C1B—C6B—C7B	−2.8 (3)	N1D—C1D—C2D—C3D	179.0 (2)
C2B—C1B—C6B—C7B	176.09 (18)	C6D—C1D—C2D—C3D	0.0 (3)
C4B—C5B—C6B—C1B	−0.2 (3)	C1D—C2D—C3D—C4D	−1.7 (3)
C4B—C5B—C6B—C7B	−178.3 (2)	C10D—O1D—C4D—C5D	−178.62 (19)
C1B—C6B—C7B—C8B	2.8 (3)	C10D—O1D—C4D—C3D	0.5 (3)
C5B—C6B—C7B—C8B	−179.1 (2)	C2D—C3D—C4D—O1D	−177.8 (2)
C1B—C6B—C7B—C11B	−176.50 (18)	C2D—C3D—C4D—C5D	1.3 (3)
C5B—C6B—C7B—C11B	1.6 (3)	O1D—C4D—C5D—C6D	179.99 (18)
C6B—C7B—C8B—C9B	−1.4 (3)	C3D—C4D—C5D—C6D	0.8 (3)
C11B—C7B—C8B—C9B	177.9 (2)	C4D—C5D—C6D—C1D	−2.4 (3)
C1B—N1B—C9B—C8B	0.2 (3)	C4D—C5D—C6D—C7D	178.43 (19)
C7B—C8B—C9B—N1B	−0.1 (3)	N1D—C1D—C6D—C5D	−176.97 (18)
C8B—C7B—C11B—O2B	−18.0 (3)	C2D—C1D—C6D—C5D	2.0 (3)
C6B—C7B—C11B—O2B	161.31 (18)	N1D—C1D—C6D—C7D	2.2 (3)
C8B—C7B—C11B—C12B	101.6 (2)	C2D—C1D—C6D—C7D	−178.76 (18)
C6B—C7B—C11B—C12B	−79.0 (2)	C5D—C6D—C7D—C8D	178.12 (19)
C16B—N2B—C12B—C13B	−64.7 (7)	C1D—C6D—C7D—C8D	−1.0 (3)
C17E—N2B—C12B—C13B	61.8 (2)	C5D—C6D—C7D—C11D	−2.6 (3)
C17B—N2B—C12B—C13B	61.8 (2)	C1D—C6D—C7D—C11D	178.22 (17)
C16E—N2B—C12B—C13B	−55.7 (6)	C6D—C7D—C8D—C9D	−0.4 (3)
C16B—N2B—C12B—C11B	61.3 (7)	C11D—C7D—C8D—C9D	−179.68 (19)
C17E—N2B—C12B—C11B	−172.19 (17)	C1D—N1D—C9D—C8D	0.6 (3)
C17B—N2B—C12B—C11B	−172.19 (17)	C7D—C8D—C9D—N1D	0.7 (3)
C16E—N2B—C12B—C11B	70.3 (6)	C8D—C7D—C11D—O2D	−13.9 (3)
O2B—C11B—C12B—N2B	−82.1 (2)	C6D—C7D—C11D—O2D	166.81 (17)
C7B—C11B—C12B—N2B	156.68 (16)	C8D—C7D—C11D—C12D	106.5 (2)
O2B—C11B—C12B—C13B	40.4 (2)	C6D—C7D—C11D—C12D	−72.8 (2)
C7B—C11B—C12B—C13B	−80.8 (2)	C17D—N2D—C12D—C13D	69.7 (2)
N2B—C12B—C13B—C14B	5.9 (6)	C16D—N2D—C12D—C13D	−52.5 (2)
C11B—C12B—C13B—C14B	−118.9 (5)	C17D—N2D—C12D—C11D	−163.67 (17)
N2B—C12B—C13B—C14E	−3.8 (5)	C16D—N2D—C12D—C11D	74.1 (2)
C11B—C12B—C13B—C14E	−128.6 (5)	O2D—C11D—C12D—N2D	−83.00 (19)
C12B—C13B—C14B—C18B	−63.9 (9)	C7D—C11D—C12D—N2D	155.25 (16)
C12B—C13B—C14B—C15B	55.7 (8)	O2D—C11D—C12D—C13D	39.7 (2)
C13B—C14B—C15B—C16B	−66.0 (10)	C7D—C11D—C12D—C13D	−82.1 (2)
C18B—C14B—C15B—C16B	52.8 (12)	N2D—C12D—C13D—C14D	−12.7 (2)
C17B—N2B—C16B—C15B	−69.2 (9)	C11D—C12D—C13D—C14D	−137.14 (18)
C12B—N2B—C16B—C15B	54.6 (11)	C12D—C13D—C14D—C15D	67.4 (2)
C14B—C15B—C16B—N2B	10.1 (12)	C12D—C13D—C14D—C18D	−51.8 (2)
C16B—N2B—C17B—C18B	56.1 (7)	C18D—C14D—C15D—C16D	65.8 (2)
C12B—N2B—C17B—C18B	−70.0 (5)	C13D—C14D—C15D—C16D	−54.9 (2)
C13B—C14B—C18B—C19B	−75.8 (11)	C17D—N2D—C16D—C15D	−56.7 (2)
C15B—C14B—C18B—C19B	165.4 (9)	C12D—N2D—C16D—C15D	65.2 (2)
C13B—C14B—C18B—C17B	54.3 (10)	C14D—C15D—C16D—N2D	−8.0 (3)
C15B—C14B—C18B—C17B	−64.4 (11)	C16D—N2D—C17D—C18D	66.0 (2)
N2B—C17B—C18B—C19B	136.3 (8)	C12D—N2D—C17D—C18D	−57.9 (2)
N2B—C17B—C18B—C14B	10.2 (9)	C15D—C14D—C18D—C19D	179.8 (2)
C14B—C18B—C19B—C20B	125.5 (9)	C13D—C14D—C18D—C19D	−61.1 (3)
C17B—C18B—C19B—C20B	0.1 (14)	C15D—C14D—C18D—C17D	−56.1 (2)
C12B—C13B—C14E—C18E	−57.5 (9)	C13D—C14D—C18D—C17D	63.1 (2)
C12B—C13B—C14E—C15E	62.9 (10)	N2D—C17D—C18D—C19D	116.7 (2)
C18E—C14E—C15E—C16E	58.4 (13)	N2D—C17D—C18D—C14D	−7.2 (2)
C13B—C14E—C15E—C16E	−62.1 (12)	C14D—C18D—C19D—C20D	−119.1 (4)
C14E—C15E—C16E—N2B	4.4 (13)	C17D—C18D—C19D—C20D	119.5 (4)
C17E—N2B—C16E—C15E	−62.1 (10)		

### Hydrogen-bond geometry (Å, º)

**Table:**

*D*—H···*A*	*D*—H	H···*A*	*D*···*A*	*D*—H···*A*
O2*A*—H2*OA*···Cl1*B*	0.84	2.23	3.0540 (18)	166
O3*A*—H3*E*···Cl2*A*	0.89 (2)	2.42 (3)	3.303 (3)	172 (4)
O3*A*—H3*F*···Cl2*B*	0.86 (2)	2.42 (3)	3.275 (3)	171 (4)
N1*A*—H1*NA*···Cl2*A*	0.88	2.06	2.935 (2)	173
N2*A*—H2*NA*···Cl1*A*	1.00	2.04	3.0226 (18)	166
C2*A*—H2*A*···O3*A*	0.95	2.51	3.414 (3)	160
C5*A*—H5*A*···Cl1*A*	0.95	2.93	3.872 (2)	172
C9*A*—H9*A*···Cl2*C*^i^	0.95	2.98	3.879 (3)	158
C11*A*—H11*A*···Cl1*A*	1.00	2.76	3.608 (2)	143
C13*A*—H13*B*···Cl2*D*^ii^	0.99	2.95	3.787 (2)	142
C15*A*—H15*A*···O2*C*^iii^	0.99	2.63	3.526 (3)	151
C16*A*—H16*A*···O2*A*	0.99	2.38	2.948 (3)	116
O2*B*—H2*OB*···Cl1*A*	0.84	2.31	3.0834 (17)	154
N1*B*—H1*NB*···Cl2*B*	0.88	2.15	3.020 (2)	168
N2*B*—H2*NB*···Cl1*B*	1.00	2.03	3.0110 (18)	168
C2*B*—H2*B*···Cl2*B*	0.95	2.72	3.479 (2)	137
C9*B*—H9*B*···Cl2*D*^iv^	0.95	2.73	3.534 (2)	142
C10*B*—H10*D*···Cl1*B*	0.98	2.86	3.606 (3)	133
C12*B*—H12*B*···Cl2*C*	1.00	2.90	3.646 (2)	132
C13*B*—H13*D*···Cl2*C*	0.99	2.78	3.559 (2)	136
C15*B*—H15*D*···O2*B*	0.99	2.51	3.041 (10)	113
C16*B*—H16*C*···O2*B*	0.99	2.45	2.942 (15)	110
C16*B*—H16*D*···Cl1*C*^v^	0.99	2.88	3.606 (16)	131
C17*B*—H17*D*···O3*C*	0.99	2.51	3.492 (3)	173
C16*E*—H16*E*···Cl1*C*^v^	0.99	2.77	3.483 (17)	129
C16*E*—H16*F*···O2*B*	0.99	2.52	3.066 (17)	115
C17*E*—H17*F*···O3*C*	0.99	2.51	3.492 (3)	173
O2*C*—H2*OC*···Cl1*D*	0.84	2.18	3.0042 (16)	166
O3*C*—H3*G*···Cl2*C*	0.88 (3)	2.51 (3)	3.381 (3)	171 (4)
O3*C*—H3*H*···Cl2*D*	0.89 (3)	2.41 (3)	3.294 (3)	168 (4)
N1*C*—H1*NC*···Cl2*C*	0.88	2.11	2.986 (2)	172
N2*C*—H2*NC*···Cl1*C*	1.00	2.02	3.0095 (18)	169
C2*C*—H2*C*···O3*C*	0.95	2.45	3.351 (3)	159
C5*C*—H5*C*···Cl1*C*	0.95	2.90	3.827 (2)	165
C11*C*—H11*C*···Cl1*C*	1.00	2.79	3.630 (2)	142
C15*C*—H15*G*···Cl1*A*^vi^	0.99	2.97	3.750 (2)	136
C15*C*—H15*H*···O2*C*	0.99	2.50	3.038 (3)	114
C16*C*—H16*H*···Cl1*A*^vi^	0.99	2.93	3.717 (2)	137
C17*C*—H17*G*···Cl1*D*^i^	0.99	2.69	3.411 (2)	130
O2*D*—H2*OD*···Cl1*C*	0.84	2.21	3.0442 (17)	172
N1*D*—H1*ND*···Cl2*D*	0.88	2.19	3.054 (2)	166
N2*D*—H2*ND*···Cl1*D*	1.00	1.99	2.9885 (18)	177
C2*D*—H2*D*···Cl2*D*	0.95	2.95	3.664 (2)	134
C5*D*—H5*D*···Cl1*D*	0.95	2.93	3.864 (2)	167
C9*D*—H9*D*···Cl2*B*^vii^	0.95	2.79	3.488 (2)	131
C10*D*—H10*K*···Cl2*A*^viii^	0.98	2.92	3.627 (3)	130
C11*D*—H11*D*···Cl1*D*	1.00	2.89	3.725 (2)	141
C13*D*—H13*G*···O3*A*^ix^	0.99	2.43	3.340 (3)	153
C16*D*—H16*I*···O2*D*	0.99	2.41	3.080 (3)	124
C16*D*—H16*J*···Cl1*B*^x^	0.99	2.82	3.653 (2)	143
C17*D*—H17*J*···Cl1*C*^viii^	0.99	2.74	3.513 (2)	135

Symmetry codes: (i) *x*+1, *y*, *z*; (ii) *x*+1, *y*−1, *z*; (iii) *x*+1, *y*, *z*−1; (iv) *x*, *y*−1, *z*; (v) *x*, *y*, *z*−1; (vi) *x*, *y*, *z*+1; (vii) *x*, *y*+1, *z*; (viii) *x*−1, *y*, *z*; (ix) *x*−1, *y*+1, *z*; (x) *x*−1, *y*, *z*+1.
